# Atmospheric observations show accurate reporting and little growth in India’s methane emissions

**DOI:** 10.1038/s41467-017-00994-7

**Published:** 2017-10-10

**Authors:** Anita L. Ganesan, Matt Rigby, Mark F. Lunt, Robert J. Parker, Hartmut Boesch, N. Goulding, Taku Umezawa, Andreas Zahn, Abhijit Chatterjee, Ronald G. Prinn, Yogesh K. Tiwari, Marcel van der Schoot, Paul B. Krummel

**Affiliations:** 1 School of Geographical Sciences, University of Bristol, Bristol, BS8 1SS UK; 2 School of Chemistry, University of Bristol, Bristol, BS8 1TS UK; 3 Department of Physics and Astronomy, Earth Observation Science, University of Leicester, Leicester, LE1 7RH UK; 4 National Centre for Earth Observation, University of Leicester, Leicester, LE1 7RH UK; 5 Atmospheric Chemistry Department, Max Planck Institute for Chemistry, Mainz, 55128 Germany; 6 Institute of Meteorology and Climate Research, Karlsruhe Institute of Technology, Karlsruhe, 76021 Germany; 7 Environmental Sciences Section, Bose Institute, Kolkata, 700054 India; 8 Center for Global Change Science, Massachusetts Institute of Technology, Cambridge, MA 02139 USA; 9 Center for Climate Change Research, Indian Institute of Tropical Meteorology, Pune, 411008 India; 10 CSIRO Oceans and Atmosphere, Aspendale, VIC 3195 Australia; 11 Present Address: National Institute for Environmental Studies, Tsukuba, 305-8506 Japan

## Abstract

Changes in tropical wetland, ruminant or rice emissions are thought to have played a role in recent variations in atmospheric methane (CH_4_) concentrations. India has the world’s largest ruminant population and produces ~ 20% of the world’s rice. Therefore, changes in these sources could have significant implications for global warming. Here, we infer India’s CH_4_ emissions for the period 2010–2015 using a combination of satellite, surface and aircraft data. We apply a high-resolution atmospheric transport model to simulate data from these platforms to infer fluxes at sub-national scales and to quantify changes in rice emissions. We find that average emissions over this period are 22.0 (19.6–24.3) Tg yr^−1^, which is consistent with the emissions reported by India to the United Framework Convention on Climate Change. Annual emissions have not changed significantly (0.2 ± 0.7 Tg yr^−1^) between 2010 and 2015, suggesting that major CH_4_ sources did not change appreciably. These findings are in contrast to another major economy, China, which has shown significant growth in recent years due to increasing fossil fuel emissions. However, the trend in a global emission inventory has been overestimated for China due to incorrect rate of fossil fuel growth. Here, we find growth has been overestimated in India but likely due to ruminant and waste sectors.

## Abstract (web summary)

India’s methane emissions have been quantified using atmospheric measurements to provide an independent comparison with reported emissions. Here Ganesan et al. find that derived methane emissions are consistent with India’s reports and no significant trend has been observed between 2010–2015.

## Introduction

Methane (CH_4_) is the second largest anthropogenic contributor to climate change after carbon dioxide (CO_2_)^1^. Because of its short lifetime of 9.8 years^2^, reductions in CH_4_ emissions will reduce radiative forcing on relatively fast timescales compared with other greenhouse gases. For this reason, CH_4_ has been identified as a target for greenhouse gas emission reduction schemes^3^; however, to do so requires an improved understanding of its main emissions sources today.

India is currently thought to have the second largest CH_4_ emissions of any country^4^, but emissions have not been quantified from the top-down using atmospheric observations from within the country and a high-resolution modelling approach. We used a combination of satellite, aircraft and surface observations (Supplementary Fig. 1) between 2010 and 2015 to quantify CH_4_ emissions from India and to investigate sources of discrepancies between the top-down derived emissions and two inventories, EDGAR_2010_ (Emissions Database for Global Atmospheric Research v4.2 FT 2010^5^) and India’s BUR (First Biennial Update Report to its National Communications^6^). This work has become possible now because we have a unique dataset comprising observations from the GOSAT (Greenhouse Gases Observing Satellite) satellite^7^, passenger aircraft observations from CARIBIC (Civil Aircraft for the Regular Investigation of the atmosphere Based on an Instrument Container)^8^ and surface measurements from Darjeeling^9^, Sinhagad^10^ and Cape Rama^11^, India. Our approach uses a high-resolution regional atmospheric transport model and a hierarchical Bayesian inverse modelling framework. Given the uncertainties in the main CH_4_ sink^12, 13^, the benefit of this regional approach is that our inversion is insensitive to uncertainties associated with CH_4_ lifetime.

We estimate average emissions over the period 2010–2015 to be 22.0 (19.6–24.3) Tg yr^−1^. These emissions are consistent with India’s reports to the United Nations Framework Convention on Climate Change (UNFCCC), but are ~ 30% smaller than the most widely used global CH_4_ inventory, EDGAR and ~ 40% smaller than previous atmospheric inversion studies over India^14^. We also find that annual emissions did not change significantly between 2010 and 2015 (0.2 ± 0.7 Tg yr^−1^), suggesting that the major CH_4_ sources, including ruminants, rice paddies, waste and fossil fuels, did not vary appreciably during this period.

## Results

### Bottom-up quantification CH_4_ emissions in India

There are significant differences in the CH_4_ emissions calculated by bottom-up inventories, further highlighting the need for top-down verification. EDGAR_2010_ estimated India’s 2010 anthropogenic emissions to be 29.6 Tg yr^−1^ with ~44%, 20%, 19% and 12% of these emissions from ruminants, waste (solid and wastewater), fossil fuels and rice paddies, respectively. As a signatory to the UNFCCC, India is required to submit National Communications. According to India’s BUR, in 2010, total emissions were 19.8 (13.6–26.0) Tg yr^−1^, with ruminants, rice, fossil fuel and waste comprising 55%, 17%, 13%, and 12%, respectively. These reported emission rates, which for many source sectors use different activity data or accounting methodologies, are substantially lower than EDGAR_2010_ emissions over the same period and are more weighted toward the agriculture sector. The largest absolute differences between 2010 EDGAR_2010_ and BUR emissions are from waste (3.46 Tg yr^−1^), fossil fuels (3.0 Tg yr^−1^) and ruminants (2.29 Tg yr^−1^).

EDGAR v4.2 FT 2012^4^ (EDGAR_2012_) reports growth of 0.4 Tg yr^−2^ in India’s CH_4_ emissions during 2010–2012. The growth rate is driven by nearly equal contributions from ruminants, waste and fossil fuels. Animal husbandry metrics from India’s Ministry of Agriculture^15^, however, show a 3% decrease in ruminant population between 2006 and 2014, which would imply decreasing emissions from the ruminant sector. If correct, this finding suggests that India may not be a major contributor to the growth in ruminant emissions recently proposed^16^.

### Emissions evaluation using atmospheric observations

**Fig. 1:**
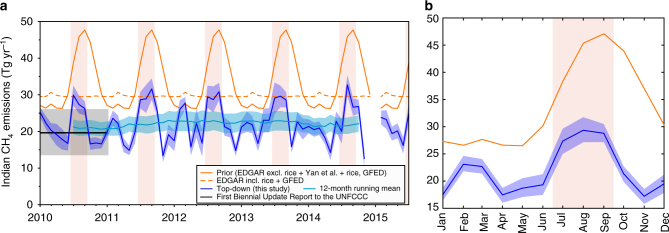
Comparison of India’s top-down CH_4_ emissions and seasonal cycle with bottom-up inventories. **a** Indian CH_4_ emissions (as Tg yr^−1^) for the prior inventories (orange, solid line) and for the top-down estimated here (dark blue line). The prior was comprised by EDGAR_2010_ (excluding rice), Yan et al.^17^ rice and GFED v3.1^18^ biomass burning. For comparison, the dashed orange line corresponds to EDGAR_2010_ (including rice) and GFED. The turquoise line and shading indicates a 12-month running mean of the top-down emissions (uncertainties assuming full correlation between months). The black line and grey shading correspond to 2010 emissions submitted to the UNFCCC (BUR) and uncertainties (based on percentage uncertainties for the year 2000, the last year for which uncertainties were published: 50% enteric fermentation, 8% rice, 125% fossil fuel, 150% waste^23^). **b** Average prior (orange) and top-down (blue) seasonal cycle. In all panels, shading corresponds to 5th–95th percentile uncertainties. The monsoon season is highlighted in pink bars

We estimated mean Indian CH_4_ emissions to be 22.0 (19.6–24.3) Tg yr^−1^ (all reported ranges are 5th–95th percentile) over the period 2010–2015 (Fig. 1a) and our estimates are consistent with the 2010 BUR emissions reported to the UNFCCC. Top-down emissions, which for India are largely comprised by anthropogenic sources, are substantially lower than those reported by global inventories (EDGAR_2010_ anthropogenic+Yan et al.^17^ rice,+Global Fire Emissions Database v3, GFED^18^) and previous atmospheric inversions over India^14^. The total growth we derived over this period 2010–2015, 0.2 ± 0.7 Tg yr^−1^ is not statistically different from zero. This is in contrast to inventories such as EDGAR_2012_ which have estimated a 0.7 Tg yr^−1^ change between 2010 and 2012 alone (uncertainties on EDGAR’s emissions are not provided). These results do not follow the same patterns as found in other high-emitting regions of the world. In the United States, a substantial underestimate was found in EDGAR emissions^19^. In addition, the trend in EDGAR_2012_ indicated decreasing USA emissions, while atmospheric observations showed flat (~ 0 ± 1 Tg yr^−2^) emissions over 2000–2014^20^. In contrast, Chinese emissions were found to be overestimated in EDGAR and increasing at 1.2 ± 0.2 Tg yr^−2^ over the period 2002–2013, a trend that is ~ 50% smaller than EDGAR’s reported trend^21, 22^. Differences in the trend in China’s emissions have been attributed mainly to the fossil fuel sector^14, 21, 22^. Compared with China, the fossil fuel sector is a much smaller component of India’s emissions (~ 16%) and therefore the difference between our top-down estimates and EDGAR’s trend is more likely to be driven by non-fossil fuel components of the inventory (e.g., ruminants or waste). China’s reported trend is ~2 ± 0.3% growth per year over 2010–2013, while we infer India’s trend to be 0.2 ± 0.6% per year over 2010–2015 (relative to the mean of the period). This finding suggests that the two countries have very different drivers of change in recent CH_4_ emissions.

The magnitude of the difference between our top-down estimate and inventories (Fig. 1 **)** remains relatively constant for most of the year (~ 9 Tg yr^−1^ during spring and autumn) but is largest in summer (~ 16 Tg yr^−1^). This suggests that the majority of the discrepancy between the top-down and prior inventories is likely due to the combination of emission sources that do not have a large seasonal cycle and those with a seasonal signature (see below). Of the sources that we do not expect to vary seasonally, ruminant and waste sectors show significant discrepancy between inventories and are therefore likely to be the most uncertain. While fossil fuel emissions are also uncertain and have been shown to be too high in China^14, 21^, this sector is a smaller component of the emissions in India, and is unlikely to explain the full discrepancy. Therefore, we propose that the seasonally invariant difference could be driven in large part by ruminant and waste sectors.

Known seasonal sources include rice and biomass burning. Natural sources such as wetlands and termites each represent only a small fraction of the total, at less than 2% of national emissions^23, 24^. However, there is evidence for additional CH_4_ emissions from sources such as open waters^25^, which may be somewhat more significant, but have not been included here owing to the limited availability of spatially and temporally explicit priors across the whole inversion domain. A double-peak was found in CH_4_ emissions, with the maximum in August (Fig. 1b) followed by a second, smaller peak in February–March. The driver of the summer maximum is likely due to monsoon season rice emissions, the time of year when the majority of the crop is grown^26^. The amplitude of the summer enhancement is approximately two-thirds of that predicted by Yan et al.^17^, which estimates annual Indian rice emissions to be 6 Tg (by integrating the emissions profile over the year). Therefore, our estimates are likely to be more consistent with annual rice emissions of ~3.9 (3.3–4.5) Tg, assuming that other sources remained relatively constant during the summer monsoon. Our rice estimate is consistent with the 2010 rice emissions in EDGAR and in the BUR. Year-to-year variability in the magnitude of the summer emissions (Supplementary Fig. 2) appears to be relatively small (8%) but is not statistically significant within uncertainties, so any changes in environmental drivers that would affect rice emissions are likely to have been smaller than the uncertainties on these emissions. The smaller winter peak indicates that there are sources that could have a seasonal signature that may not be represented well by inventories. This could be due to winter rice, which comprises 14% of total rice production in India^26^ or wintertime increases in fossil fuel emissions^23^. The latter is primarily comprised by biomass burning of dung cakes and fuel wood from the residential sector^27^. Large-scale biomass burning, based on satellite data, represents < 5% of the total emissions in this region apart from slightly increased emissions (10% of total) during March and is less likely to be the source of this winter peak.

**Fig. 2:**
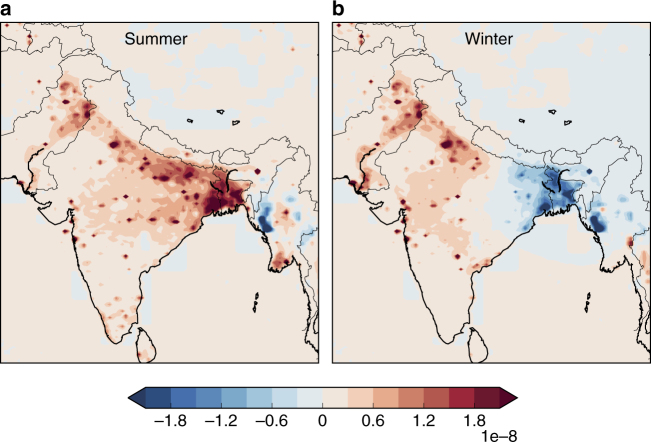
Differences in emissions between seasons. **a** Difference between the average summer (June–Sept) or **b** average winter (Jan–Feb) peak emission periods, and the average of spring/autumn minimum emission periods (March–May, October–December) in g m^−2^ s^−1^. Scaling factors to the prior flux map for each month were estimated for ~40 spatial basis functions within this domain; the prior map was scaled up or down by this factor for each basis function

**Fig. 3:**
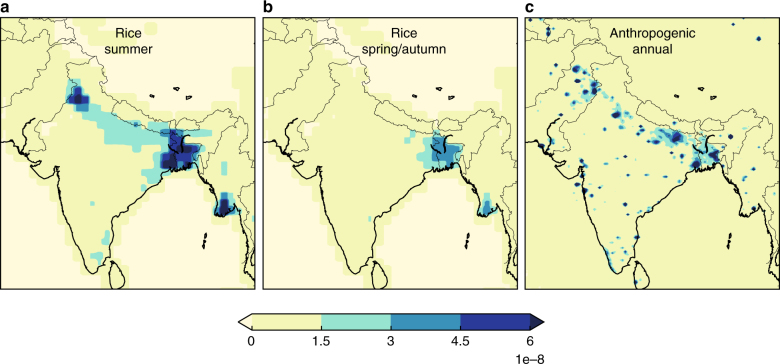
Prior emissions in g m^−2^ s^−1^ by source sector. **a** Yan et al.^17^ average rice emissions for June–September. **b** Yan et al.^17^, average rice emissions for March–May and October–December. **c** EDGAR v4.2FT2010 anthropogenic emissions excluding rice and ruminants (diffuse sources)

One utility of high-resolution modelling is that spatial maps can be used to identify patterns of change to provide an additional fingerprint for source identification. An analysis of the difference between summer or winter emissions (i.e., during peak emission periods) and the average of the spring and autumn emissions (i.e., minimum emissions periods) show where peak emissions are enhanced relative to the minima for the year. The main summer peak shows enhancement in the primary rice growing regions of the Indo-Gangetic Plains and North-eastern India (Figs. 2a and 3a). In contrast, the secondary winter peak during February–March corresponds with increased emissions from populated areas and a decrease in the northeast relative to spring/autumn (Fig. 2b). This pattern suggests that the winter peak is not likely due to winter rice but to an anthropogenic source occurring in populated areas (Fig. 3c). Additional information using other tracers would be required to specifically identify the sector associated with this increase. The small decrease in the northeast is due to the difference relative to small spring/autumn rice emissions in the inventory (isolated to Northeast India) (Fig. 3b).

Emission maps and their uncertainties are shown in Supplementary Figs. 3–5. The posterior uncertainty maps in Supplementary Fig. 6 indicate that the data has the greatest constraint on emissions from the Indo-Gangetic Plains of Northeast India, the region with the largest emissions, but the majority of the country is well-sampled with the satellite data.

## Discussion

Our results imply little growth in Indian CH_4_ emissions during the period 2010–2015. Robust evaluation of national emissions and their trends will be vital to the success of international climate agreements, particularly in light of large uncertainties on some sectoral components of inventory estimates. Our results here are consistent with India’s BUR and should go some way to enhance confidence in India’s CH_4_ inventory. In contrast, our estimates are 30% smaller than global inventories such as EDGAR. These findings demonstrate the need for more robust bottom-up accounting, using country-specific data and more advanced accounting methodologies. They also highlight the value of top-down evaluations of emissions inventories for regions of the world that are critical for global climate and policy, but like India, are under-studied and are therefore poorly quantified contributors to climate change.

## Methods

### Observations

We used data from satellite, aircraft and surface sources over the period 2010–2015. These data come from the GOSAT satellite, CARIBIC aircraft and Darjeeling (DJI), Cape Rama (CRI) and Sinhagad (SNG), India surface stations. A map of site locations and typical CARIBIC and GOSAT sampling is provided in Supplementary Fig. 1. GOSAT dry air column-averaged mole fractions (XCH_4_) and the percentage difference between three chemical transport models used to derive XCH_4_ are found in Supplementary Figs. 7, 8. In addition, a comparison between CO_2_ observations and one of the chemical transport models used in the generation of XCH_4_ is found in Supplementary Fig. 9. A description of each dataset is found in Supplementary Note 1.

### Generation of NAME sensitivity maps

We used a model to provide the link between fluxes and atmospheric concentrations and this allows us to use measured concentration data to infer fluxes. Here, we used a Lagrangian Particle Dispersion Model (LPDM) to quantify the sensitivity of changes in mole fractions to (i) changes in surface emissions and to (ii) changes in boundary conditions at the edges of the LPDM domain. These were calculated using the UK Met Office model NAME (Numerical Atmospheric dispersion Modelling Environment 3) model^28^. NAME was driven by the Unified Model’s South Asian Model meteorology at 12 km horizontal resolution (until 2014) and the Unified Model Global Meteorology at 16 km after 2014, for 70 vertical levels and at three-hourly temporal resolution^9, 29^. While transport processes at finer scales, such as convection, are parameterized within NAME, the effects of flux processes occurring at small-scales (<10 km) will not be resolved by the model. However, it is important to note that it is not the intention of a study such as ours to resolve these small-scale fluxes. Instead, we aim to interpret larger-scale fluxes (>100 km). This is possible because the atmosphere integrates CH_4_ fluxes over regional scales through advection and mixing. It is these large-scales that we primarily observe with atmospheric concentration data, in contrast to for example, flux data (e.g., eddy covariance or chamber measurements), which observe small-scale processes (typically <1 km). The inversion domain spanned from 55–110°E to 6–48°N and up to 19 km vertically. Output resolution was 0.352×0.234°. Model sensitivities were derived with respect to the surface and this was defined to be 0–40 metres above ground level. All simulations were run for a maximum of 30 days backwards in time but the majority of particles exited the domain prior to this time. The time and location that particles left the domain were recorded to provide the sensitivity to boundary conditions. NAME has been used extensively to model atmospheric concentrations of long-lived greenhouse gases^30–33^ and was one of the participating models in a regional flux inversion comparison exercise^34^.

### Surface and aircraft observations

For each measurement point, 2000 particles were released per hour and this number was chosen to ensure a high signal-to-noise in the resulting footprints. Release rates used in other LPDM studies have ranged between 500 and 10,000 particles per hour^19, 35^. For DJI, the release height was chosen to be 500 m.a.g.l. to account for any unresolved topography at the site. This release height is the midpoint between the true surface height and the model height. This approximation has been used at other mountain sites^36^. Observations were averaged into 3 h periods. For flask sites CRI and SNG, the release height was 10 magl, the height of sampling on the towers.

The relationship between concentrations and emissions can be expressed through Eq. (1):

1 $${\bf{y}} = {\bf {H}}{\bf{x}}$$

where **y** is a vector of *m* simulated mole fractions, **H** is a matrix of sensitivities of dimension *m* x *n* with *n* being the resolution of the underlying basis function in the inversion and **x** is a vector of *n* unknowns. As discussed further below, an ensemble of inversion grids was sampled in the inversion such that the dimension *n* was not fixed a priori.

### Satellite observations

Dry-air column-averaged mole fraction, XCH_4_, was derived based on 20 vertical levels for the CH_4_ prior, averaging kernel and pressure weights. NAME simulations were performed for each vertical level. For computational efficiency, the following method was employed. Particles were released at a rate of 1000 per hour for levels 1–8 and at 100 per hour for levels 8–17. Above level 8, there was no significant sensitivity to the surface and these particles were required only to provide the sensitivity of each level to boundary conditions. In this way, the computational time required to compute the sensitivity map for each retrieval was kept to a minimum.

Above some maximum level (maxlev), which was usually level 17 for this domain, the column height exceeded the height of the NAME model (set here at 19 km) and therefore, concentrations at these levels were assumed to be the concentrations from the prior CH_4_ field. This assumption is discussed further in Supplementary Note 2.

Equation (2) governs the conversion of model mole fractions generated at each level to a column value that could be compared to the satellite observation. For each retrieval occurring at time index *t*:

2 $${{\rm XCH}_4^{\rm model}}{|_t} = \mathop {\sum}\limits_1^{20} {{p_i}\left[ {{A_i} \cdot {{\rm CH}_{4,i}^{\rm model}} + \left( {1 - {A_i}} \right) \cdot {{\rm CH}_{4,i}^{\rm prior}}} \right]}$$

where *p*_*i*_ is the pressure-weighting at level *i* as discussed in O’Dell et al.^37^. *A*_*i*_ is the averaging kernel at level *i*, CH_4,i_^prior^ is the prior mole fraction at level *i* and CH_4,i_^model^ is the model mole fraction at level *i* ^38^. For NAME, CH_4,i_^model^ is made up of a signal due to regional emissions and a signal due to boundary conditions (Eq. (3)).

3 $$X{{\rm CH}_{4,i}^{\rm model}}{|_t} = {{\bf{h}}_{\bf{i}}} \cdot {\bf{q}} + {{\bf{h}}_{{\bf{b}},{\bf{i}}}} \cdot {\bf{b}}$$

where **h**_**i**_ is the NAME surface sensitivity derived for level *i*, **q** is a vector of emissions, **h**_**b,i**_ is the sensitivity of level *i* to boundary conditions and **b** is a vector of boundary conditions.

Because the second term of Eq. (2) is known before the inversion, this term can be removed from both XCH_4_^model^ *|*_*t*_ and the observation. To account for the mole fractions above maxlev, the weighted contribution of mole fractions from the prior model above this level was also removed from XCH_4_^model^ *|*_*t*_ and the observation. This generates XCH_4,pert_^model^ for each retrieval *t* (Eq. (4)).

4 $${{\rm XCH}_{4,\rm pert}^{\rm model}}{|_t} = {{\rm XCH}_4^{\rm model}}{|_t} - \mathop {\sum }\limits_1^{{\rm maxlev}} {p_i}\left[ {\left( {1 - {A_i}} \right) \cdot {{\rm CH}_{4,i}^{\rm prior}}} \right] - \mathop {\sum }\limits_{{\rm maxlev}}^{20} {p_i}\left[ {{{\rm CH}_{4,i}^{\rm prior}}} \right]$$

Equation (4) thus reduces to the following:

5 $${{\rm XCH}_{4,\rm pert}^{\rm model}}{|_t} = \mathop {\sum }\limits_1^{{\rm maxlev}} {p_i} \cdot {A_i} \cdot ({{\bf{h}}_{\bf{i}}} \cdot {\bf{q}} + {{\bf{h}}_{{\bf{b}},{\bf{i}}}} \cdot {\bf{b}})$$

Equation (5) can be written in a form for all retrievals (i.e., over all times) (Eq. (6)).

6 $${\bf{XCH}}_{4,{\bf{pert}}}^{{\bf{model}}} = {\bf{H}}{\bf{x}}$$

Because **q** and **b** are fields that are independent of level, for row *t* in **H**, the columns are made up of the sum over levels of **h**_**i**_ and **h**_**bi**_ (weighted by averaging kernel and pressure weight). **x** is a vector stacked with **q** and **b**. With these modifications to the equations representing modelled mole fractions from the satellite, Eqs. (1) and (6) are now in the same form and can be compressed into a single equation for all observations (surface, aircraft and satellite), but with the GOSAT observations in the data vector being the modified values.

Supplementary Fig. 10 shows an example NAME footprint generated for a single GOSAT observation. This figure indicates that each GOSAT observation is most sensitive to surface emissions within ~ 100 km.

### Boundary conditions

A priori information about boundary conditions were taken from the global Eulerian model MOZART (Model for OZone And Related Tracers)^39^, output at 1.9×2.5°and monthly resolution, by mapping the exit location of particles to the mole fractions represented on curtains to the NAME domain (Supplementary Fig. 11). MOZART was run using global emission inventories (EDGAR v4.2 for anthropogenic emissions^40^, Bloom et al.^41^ for wetlands and rice, Fung et al.^24^ for other natural sources and GFED v3.1 for biomass burning emissions^18^) and were not optimised with any measurement information, thus maintaining independence between this inversion and the prior.

The boundary condition curtains were decomposed into a set of basis functions, adjustments to which were also estimated in the inversion. These terms included: an offset shifting the entire field up or down uniformly, a scaling to multiply the entire field, a scaling to the North-South gradient, a scaling to the East-West gradient, and a scaling to the stratospheric gradient. In total, five boundary condition terms were estimated per month. Because these adjustments to the MOZART boundary conditions were estimated in the inversion, uncertainties in the CH_4_ lifetime, which were used to generate the prior, become insignificant in the posterior solution.

### Trans-dimensional Markov Chain Monte Carlo inversion

Bayesian inversions are a statistical tool used to develop posterior beliefs by refining our prior knowledge with the assimilation of data. Uncertainties in the prior and the data indicate the relative weights in each set of information. Data is never itself altered. Improvements made to the emissions field can be assessed by comparing the posterior mole fractions (emissions run through the atmospheric transport model) with the original data to see how the fit has improved over the prior.

LPDM sensitivities were applied in a hierarchical Bayesian inverse framework using trans-dimensional Markov chain Monte Carlo (TDMCMC) to estimate monthly fluxes, boundary conditions, an offset term representing differences between satellite/calibrated data and uncertainty parameters. The hierarchical Bayesian methodology followed that described in Ganesan et al.^42^. To reduce the subjectivity of incorrectly specified uncertainties, hyper-parameters were included consisting of model error, prior emissions error and hyper-parameters that govern the number and spatial distribution of unknowns in the underlying flux field. These hyper-parameters, each with their own PDFs that were sampled in the inversion, allowed us to explore any uncertainties in uncertainties. This allows us to better account for random uncertainties in the system, which traditionally have been fixed but largely subjective quantities. The posterior PDF includes the effect of these unknown uncertainties and are therefore more representative of the true unknowns in the system. Systematic errors, which may arise from, for example, structural errors in the model were not quantified through this method. Quantifying such uncertainties is an area of on-going research. The MCMC approach also allowed us the flexibility to use any probability density function (PDF), without needing to impose Gaussian distributions, a limitation of many traditional inversions.

We built on this approach following advancements made in Lunt et al.^43^, by also including hyper-parameters that govern the number and spatial distribution of unknowns in the underlying flux field (i.e., the underlying inversion grid which has typically been subjectively chosen)^43^. In this trans-dimensional system, which refers to the fact that the dimension of the problem is also characterised in the inversion, we were also able to account for uncertainties in the underlying model decomposition. Fluxes were not inferred at the resolution of the meteorological drivers running NAME (12 km); instead, the inversion domain was decomposed into a set of basis functions (grid cells that are grouped into larger regions), and it was different configurations of this grouping that was also sampled in the inversion. Lunt et al.^43^ have shown that this algorithm explores an ensemble of inversion grids that cluster around solutions that maintain the most simplicity to explain the data, while minimising aggregation error. While the Lunt et al.^43^ method updated one emissions element per step, the code used in this work was modified to update ten random elements per step for improved efficiency.

In this framework, each iteration samples a new value of parameters from the PDFs, which are characterised by hyper-parameters. We assumed a lognormal emissions PDF to prevent non-physical negative solutions and the shape of this PDF was governed by hyper-parameters for the median and standard deviation. We have chosen PDFs of our hyper-parameters to be as uninformative as possible (i.e., a uniform distribution with a broad range) to allow flexibility for the data to drive the solution. For the emissions PDF, we assumed the median to lie in a range of 0.1–10 times the inventory value and for the standard deviation to lie in a range of 5–500% of the median value. We assumed a Gaussian model-measurement discrepancy PDF with the s.d. lying in a uniform distribution with range 5–200 ppb. Measurement uncertainty was treated as a fixed, known quantity, comprised by instrumental precision and variability within the averaging period and added to the hyper-parameter model error in quadrature. Model error was assumed to be uncorrelated based on findings from Ganesan et al.^33^ which showed that for networks of this type, measurements averaged over 3 h and 100 km were largely uncorrelated.

Fluxes, boundary conditions and hyper-parameters were estimated for each month separately. For each month, we sampled 100,000 times with an additional 100,000 burn-in samples to remove any memory of the initial state. We thinned these samples to reduce redundant (auto-correlated) information in the chain by storing every 100th sample. The acceptance ratio for parameters ranged between 0.2 and 0.5, which is recommended for optimal mixing^44^. This was achieved by varying the step sizes of each boundary condition element individually, each fixed region emissions element individually and for all the variable emission regions through a single parameter. A single step size was used for emissions from all the variable regions since the dimensionality changes with each step. The acceptance ratio was then calculated as the average of all emissions elements in the trans-dimensional domain. Tuning step sizes helps to achieve optimal mixing so that convergence is reached with greatest efficiency. This was done automatically by adjusting the step size every 500 iterations through the burn-in period, calculating the acceptance ratio (individually for boundary condition and fixed emissions elements and as an average for the variable emissions elements), and then increasing/decreasing the step size if the acceptance ratio of the previous 500 iterations was not in the range 0.2–0.5. Step sizes then remained fixed after the burn-in period. Though we only tuned one step size for all the variable regions, we assessed convergence for each grid cell (by disaggregating the emissions element to the native grid at each step) in the trans-dimensional domain by performing Geweke’s diagnostic. This was done by quantifying the mean of the first 10% and the last 50% of the chain. Convergence was reached when these two values were not statistically different. The number of parameters estimated each month was ~50. This is comprised of five boundary condition elements, one ‘offset’ term representing differences between the satellite and calibrated data, four parameters describing emissions in fixed regions outside of the trans-dimensional area, and ~40 emissions parameters estimated within the trans-dimensional area. The number of regions estimated is shown by the histogram in Supplementary Fig. 12, which represents the frequency of the number of regions in the 100,000 samples. This is an example for 1 month but was typical for most months. Uncertainty characteristics of the inversion are discussed further in Supplementary Note 2.

Comparison of the prior and posterior mole fractions for each site is found in Supplementary Figs. 13–30.

### Sensitivity analyses

We performed five sets of sensitivity studies. (1) We quantified the effect of using a prior without a seasonal cycle to assess the influence of the prior on the top-down emissions (Supplementary Fig. 31). (2) To assess the accuracy of the CO_2_ field used to generate XCH_4_, we analysed both the variability in three CO_2_ chemical transport models/inversion setups as well as the overall fit of the CO_2_ model to independent (i.e., not assimilated) CO_2_ data. The XCH_4_ retrievals used in this study were derived using the median XCO_2_ from an ensemble of three models: MACC-II, Carbon-Tracker and GEOS-Chem^7^. We explored the effect of perturbing XCH_4_ to higher and lower values based on the range of XCO_2_ for each sounding (Supplementary Fig. 32). (3) We quantified the effect of randomly re-sampling the GOSAT soundings to assess the effect of the GOSAT track on the derived emissions. In each month over the period, the inversion was re-run using a random selection of 200 samples with replacement (Supplementary Fig. 33). (4) We assessed the effect of using GOSAT data alone vs. the full set of surface, aircraft and satellite observations to understand any differences associated with sampling (Supplementary Fig. 34). (5) We quantified the effect of errors in the MOZART model’s simulation of the stratosphere on derived emissions (Supplementary Fig. 35). Sensitivity studies are discussed further in Supplementary Note 3.

### Code availability

Code will be made available upon request by contacting Anita Ganesan. The TDMCMC inversion code was written in Fortran 90 and additional components are available for setting up the simulation in Python 2.7.

### Data availability

Data can be accessed by contacting data leads: Robert Parker for GOSAT, Andreas Zahn for CARIBIC, Anita Ganesan for Darjeeling, Paul Krummel for Cape Rama and Yogesh Tiwari for Sinhagad.

## Electronic supplementary material

Supplementary Information
