# Supplementary material for: Atmospheric observations show accurate reporting and little growth in India’s methane emissions
Source: Nat Commun. 2017 Oct 10;8:836. doi: 10.1038/s41467-017-00994-7 (PMC5635116; doi:10.1038/s41467-017-00994-7)
Supplement: Supplementary file 1 — Supplementary Information [file 41467_2017_994_MOESM1_ESM.pdf]

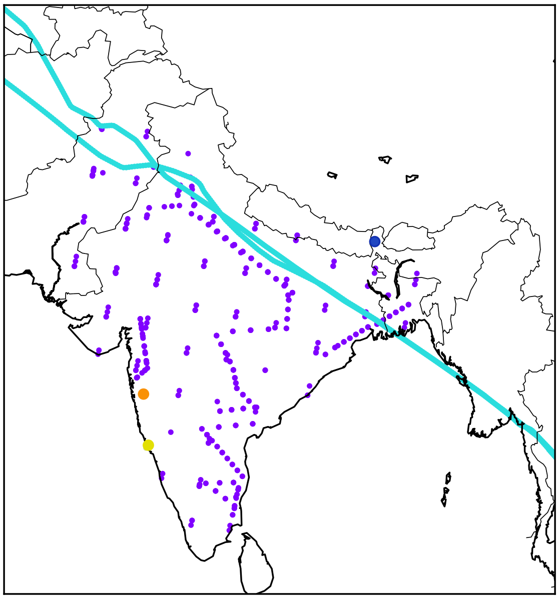

Supplementary Figure 1: Map of data sources used in this study (not all simultaneously). Typical monthly coverage from GOSAT retrievals (purple), CARIBIC flight path (light blue), Darjeeling India (dark blue), Cape Rama India (yellow), Sinhagad India (orange).

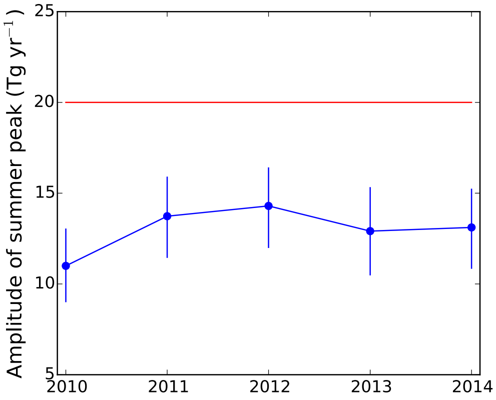

Supplementary Figure 2: Amplitude of the summer peak in Tg yr^{-1}, defined as the average of June-September emissions minus the average of April and October emissions. Red line corresponds to the the Yan et al., (2009)[1], rice climatology used in the prior and blue line corresponds to the top down with 5th-95th percentile uncertainties.

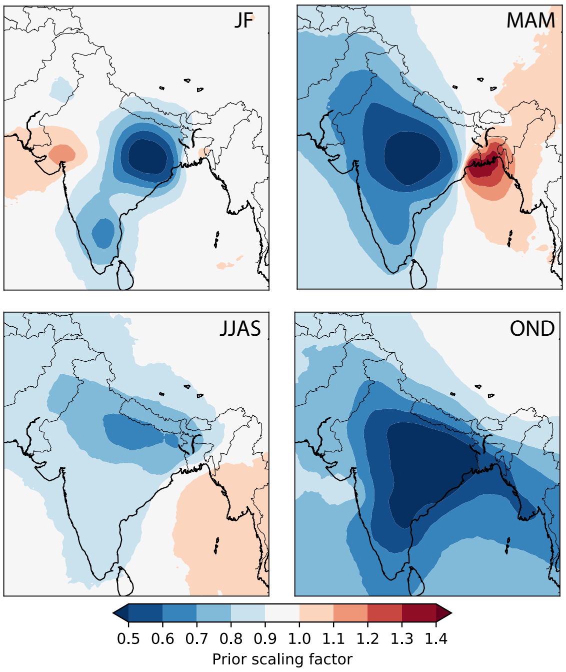

Supplementary Figure 3: Top-down estimated scaling factors of the prior for each season (unitless). Scaling factors were estimated for approximately 40 spatial basis functions within this domain for each month; the prior map was scaled up or down by this factor for each basis function.

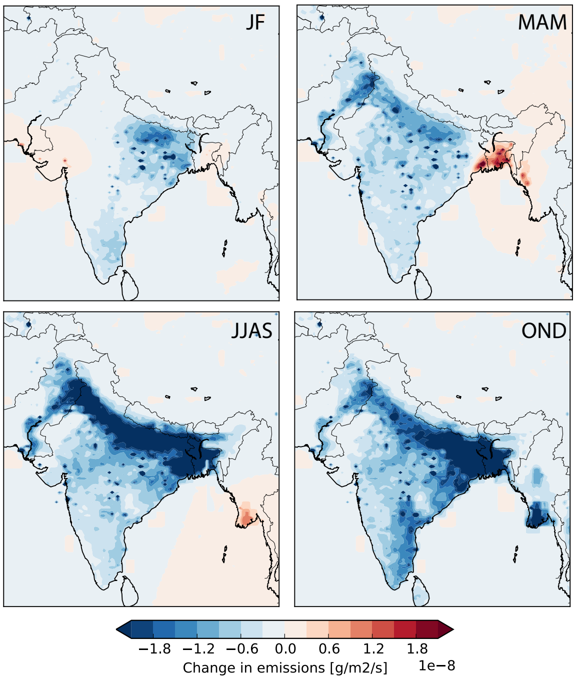

Supplementary Figure 4: Difference between top-down and prior emissions for each season in g m^{-2} s^{-1}. Seasons have been defined by the India Meteorological Department classifications: Winter (Jan-Feb), Pre-monsoon (Mar-May), Monsoon (June-Sep), Winter (Oct-Dec). Scaling factors to the prior flux map for each month were estimated for approximately 40 spatial basis functions within this domain; the prior map was scaled up or down by this factor for each basis function. Maps of scaling factors for each season are shown in Supplementary Figure 3.

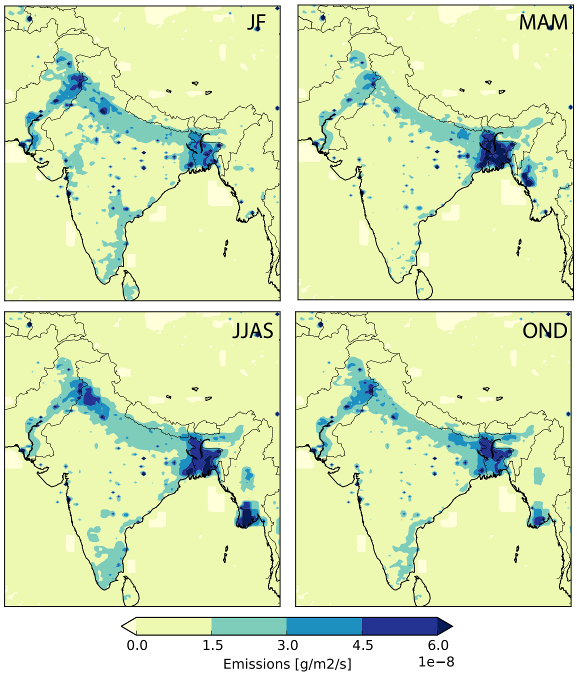

Supplementary Figure 5: Top-down emissions for each season in g m^{-2} s^{-1}. Scaling factors to the prior flux map for each month were estimated for approximately 40 spatial basis functions within this domain; the prior map was scaled up or down by this factor for each basis function. Maps of scaling factors for each season are shown in Supplementary Figure 3.

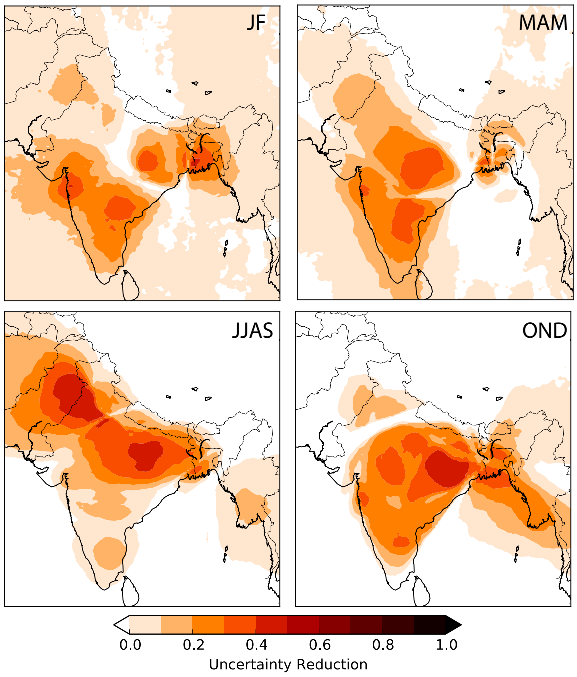

Supplementary Figure 6: Average ‘top-down’ seasonal emissions uncertainty reduction (unitless), defined as the difference between the posterior uncertainty and the prior uncertainty (sampled through hyper-parameter PDFs). Uncertainties were calculated as the ratio of the 5th-95th percentile range to the mean emissions.

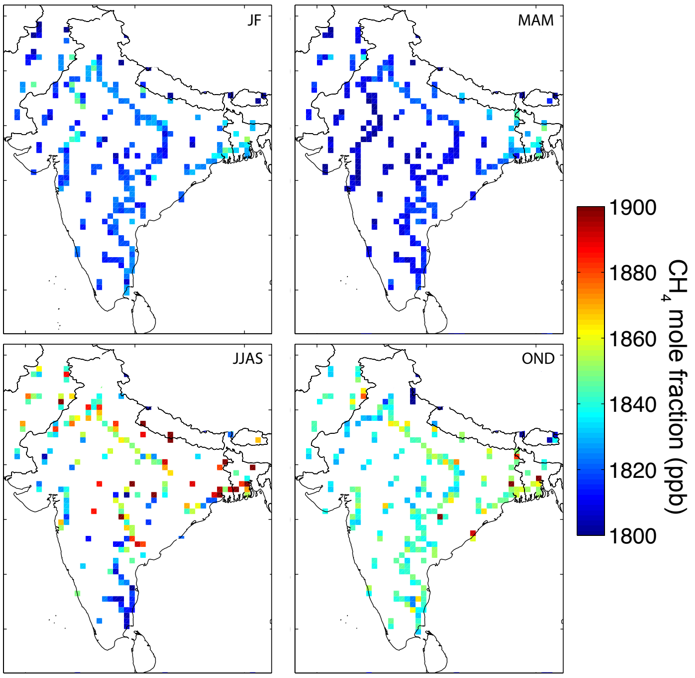

Supplementary Figure 7: Average GOSAT XCH_4 mole fractions (ppb) over 2012 for each season. Due to increasing mole fractions over time and slightly different sampling each year, only 2012 is shown for clarity, rather than an average over 2010-2015.

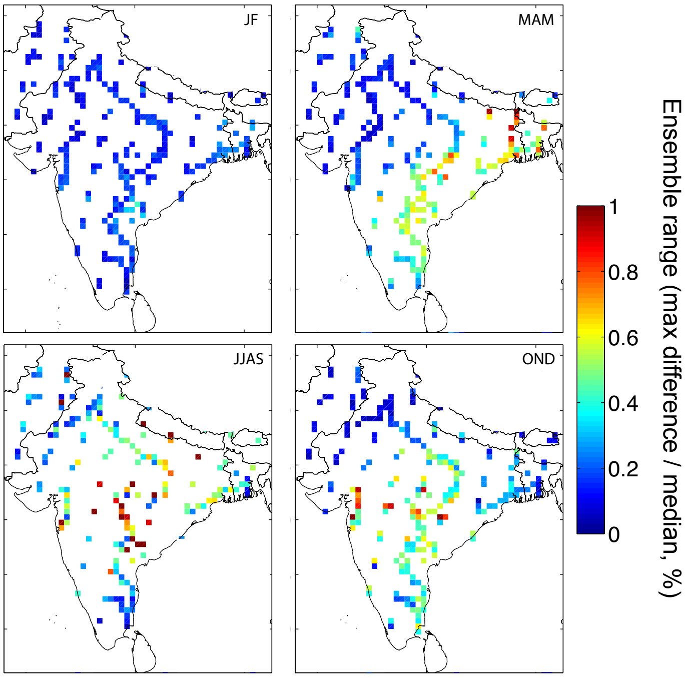

Supplementary Figure 8: Percentage difference between three chemical transport models/inversion methods for XCO_2, which was used to derive proxy XCH_4. Metric is defined as the maximum difference between ensemble members and the median model divided by the median model. Due to increasing mole fractions over time and slightly different sampling each year, only 2012 is shown for clarity, rather than an average over 2010-2015.

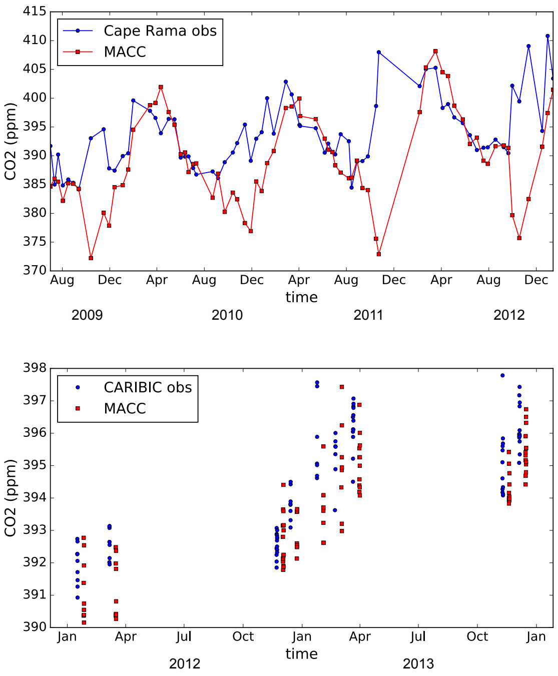

Supplementary Figure 9: Comparison between MACC-II model CO_2 and CO_2 observations (ppb) from (top) Cape Rama, India (CRI) and (bottom) CARIBIC. Note: CARIBIC model CO_2 (red squares) has been offset slightly in time for clarity.

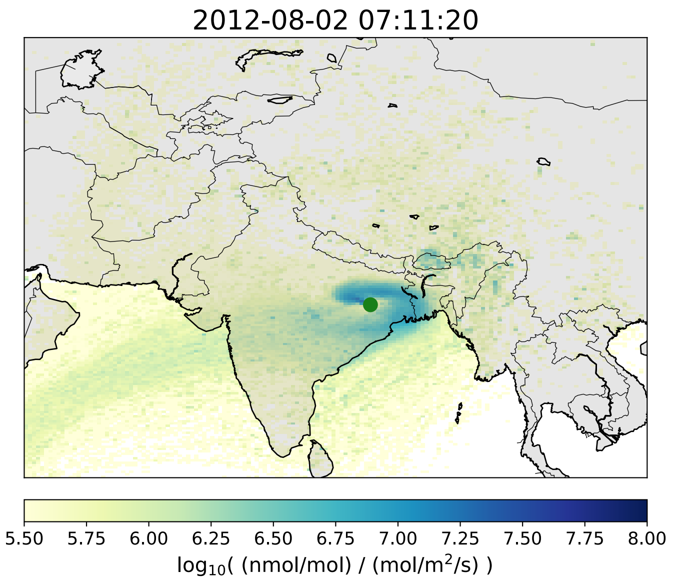

Supplementary Figure 10: Example of a NAME footprint (surface sensitivity) map for a single 0.5 degree averaged GOSAT measurement (log[(nmol mol^{-1})/(mol m^{-2} s^{-1})]). Note the logarithmic scale.

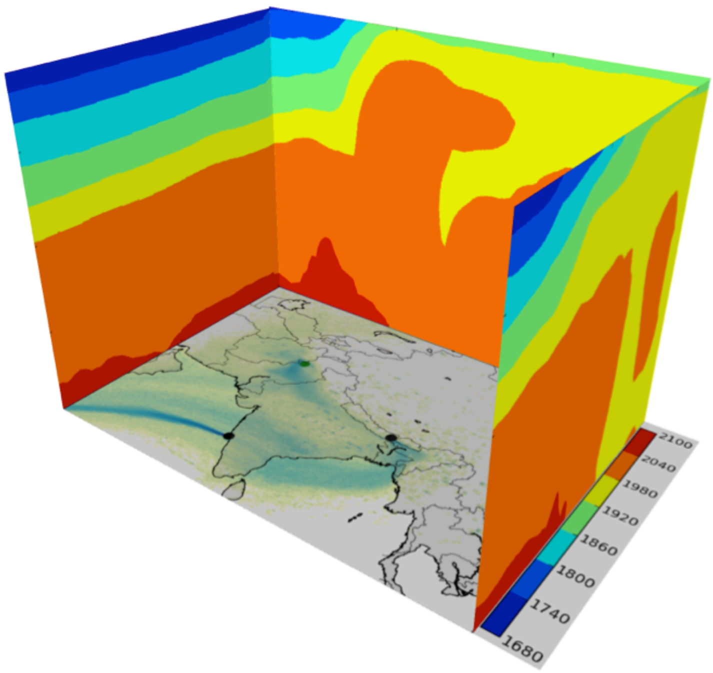

Supplementary Figure 11: MOZART curtains (CH_4 mole fraction in ppb) around the NAME LPDM domain. Curtains are extracted at 1.9^{o}x2.5^o and monthly resolution. Figure represents one snapshot in time.

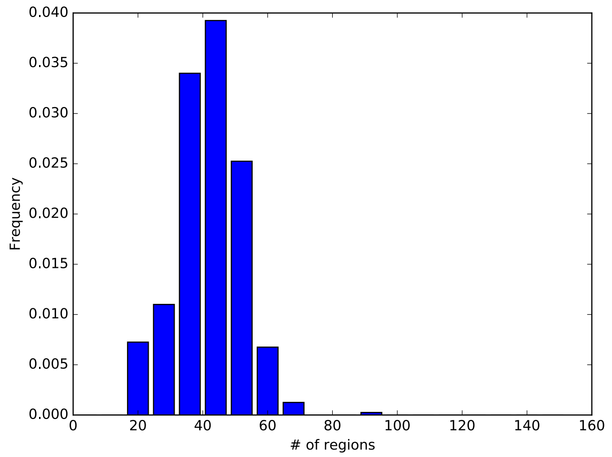

Supplementary Figure 12: Histogram of the number of regions sampled in the MCMC for a typical month. Regions represent the flux field decomposition (i.e. basis functions) used in the inversion. The number and spatial distribution of these regions vary at each iteration. The mean number of regions estimated is approximately 40.

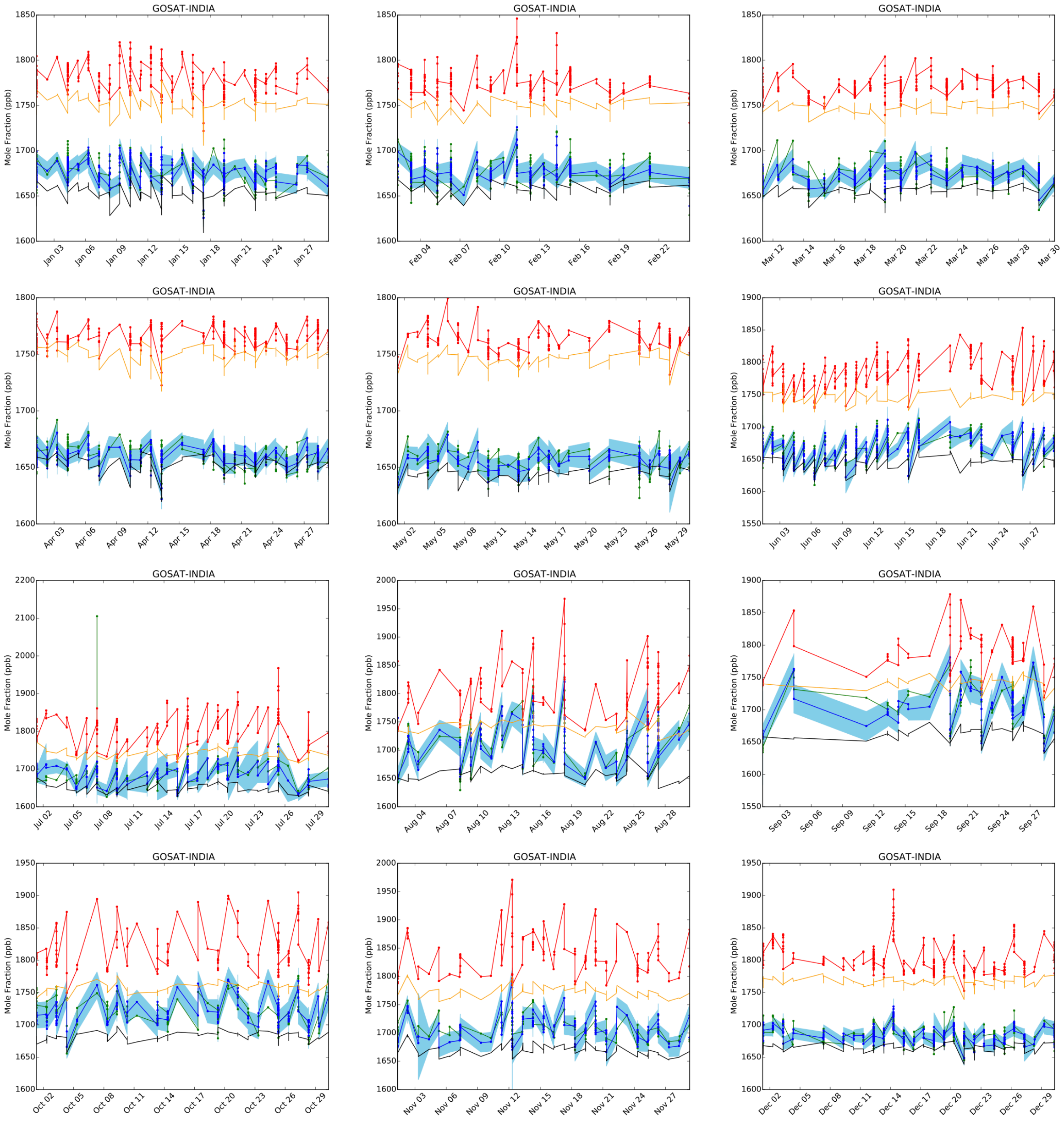

Supplementary Figure 13: GOSAT observation and model comparison (ppb) for 2010. Prior (red) and posterior (blue) mole fractions and prior (orange) and posterior (black) boundary conditions. Observations are shown in green with blue shading indicating the 5-95th percentile range of the model-measurement error derived in the inversion. Mole fractions represent the term $\boldsymbol{XCH_{4,pert}^{model}}$ in equation (7)

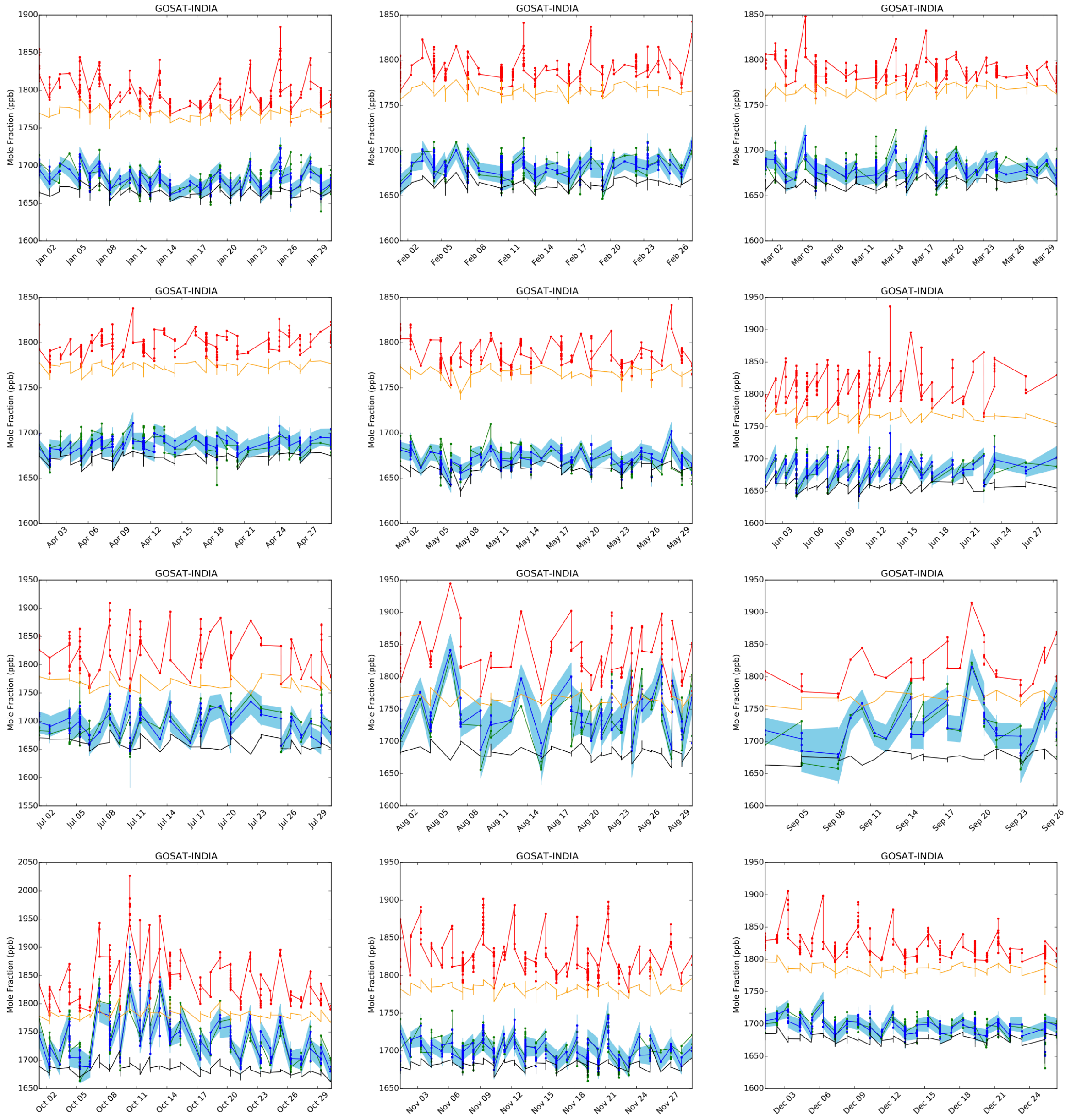

Supplementary Figure 14: GOSAT observation and model comparison (ppb) for 2011. Prior (red) and posterior (blue) mole fractions and prior (orange) and posterior (black) boundary conditions. Observations are shown in green with blue shading indicating the 5-95th percentile range of the model-measurement error derived in the inversion. Mole fractions represent the term $XCH_{4,pert}^{model}$ in equation (6).

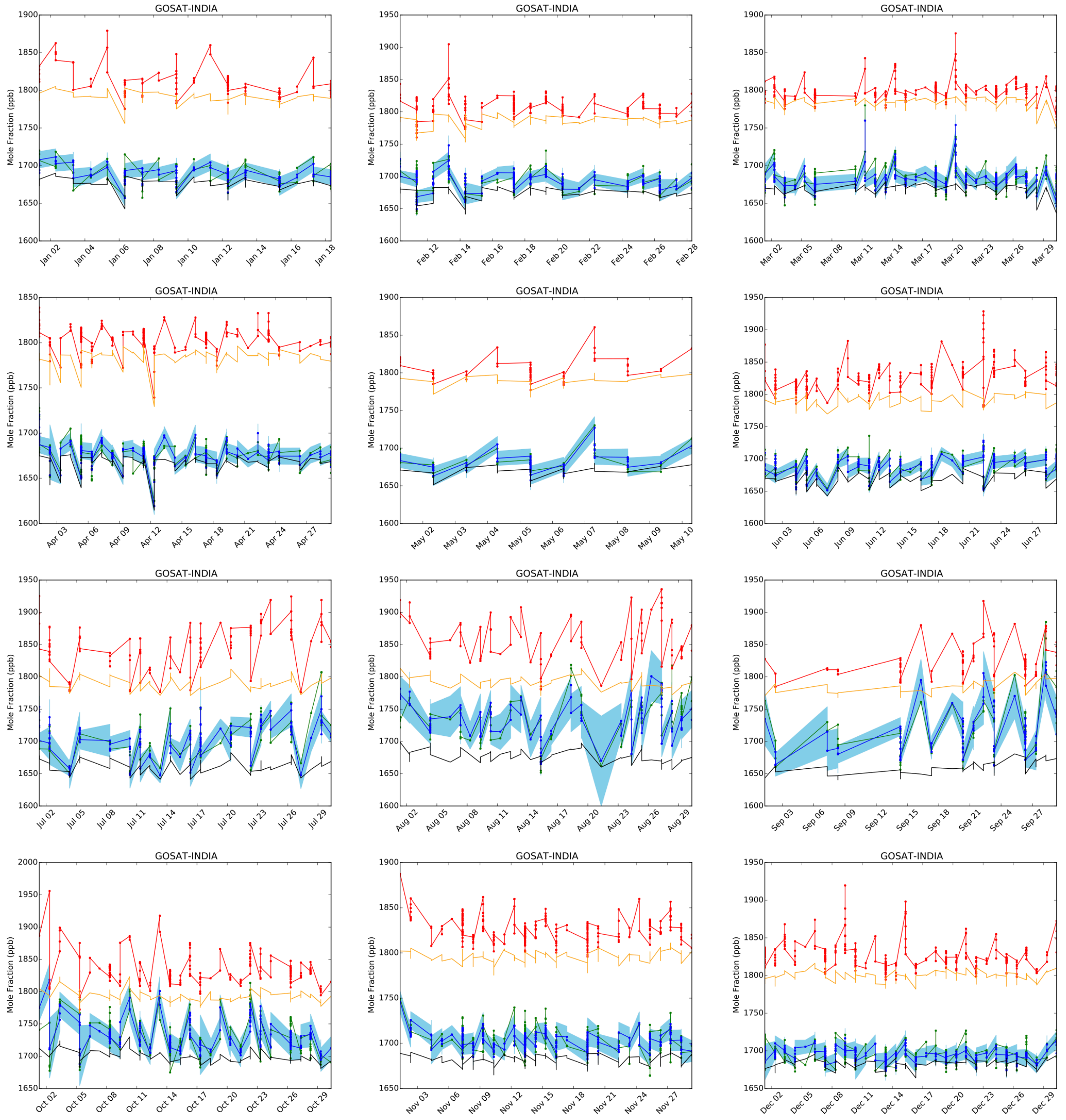

Supplementary Figure 15: GOSAT observation and model comparison (ppb) for 2012. Prior (red) and posterior (blue) mole fractions and prior (orange) and posterior (black) boundary conditions. Observations are shown in green with blue shading indicating the 5-95th percentile range of the model-measurement error derived in the inversion. Mole fractions represent the term $\boldsymbol{XCH_{4,pert}^{model}}$ in equation (6).

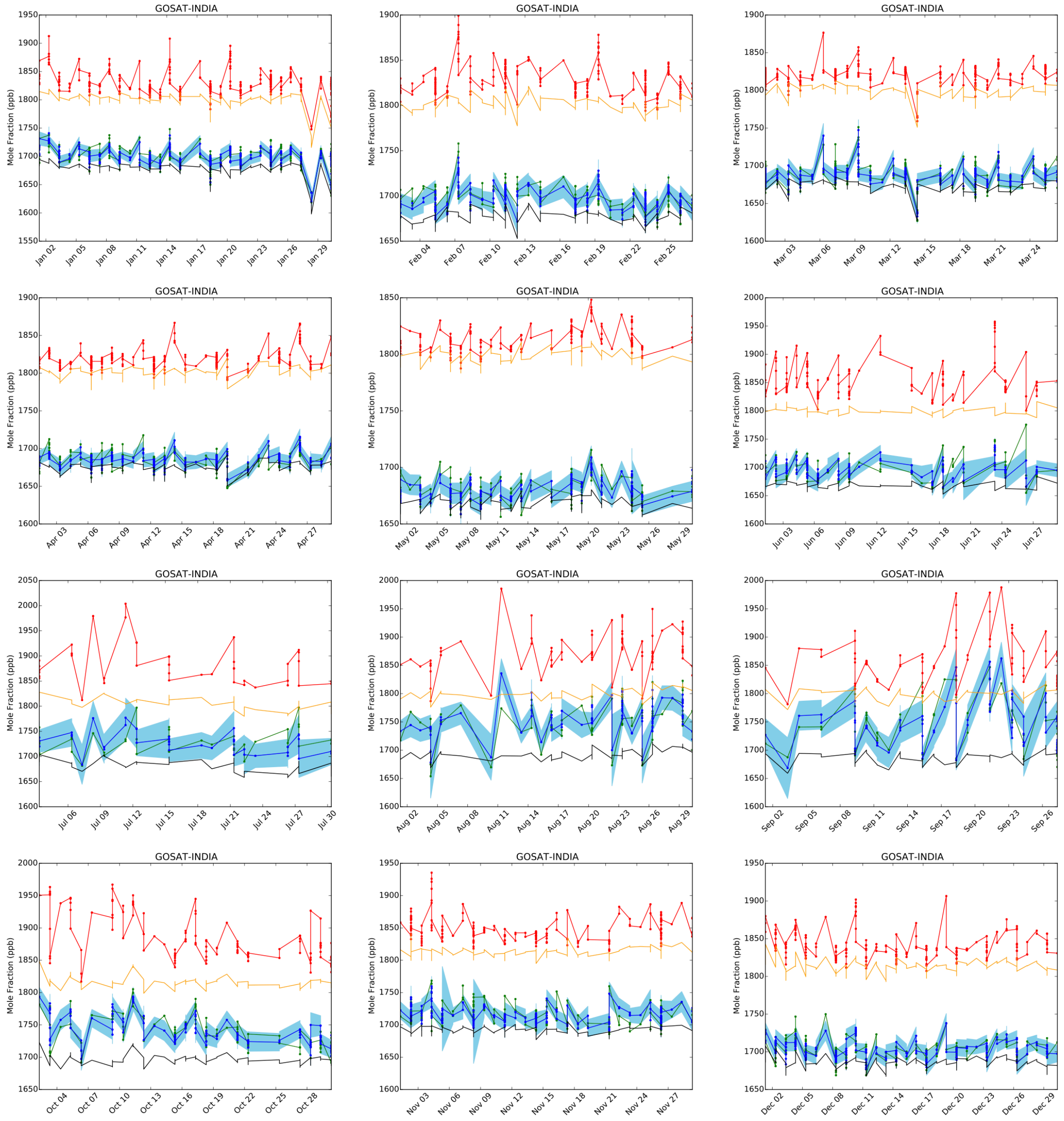

Supplementary Figure 16: GOSAT observation and model comparison (ppb) for 2013. Prior (red) and posterior (blue) mole fractions and prior (orange) and posterior (black) boundary conditions. Observations are shown in green with blue shading indicating the 5-95th percentile range of the model-measurement error derived in the inversion. Mole fractions represent the term $\boldsymbol{XCH_{4,pert}^{model}}$ in equation (6).

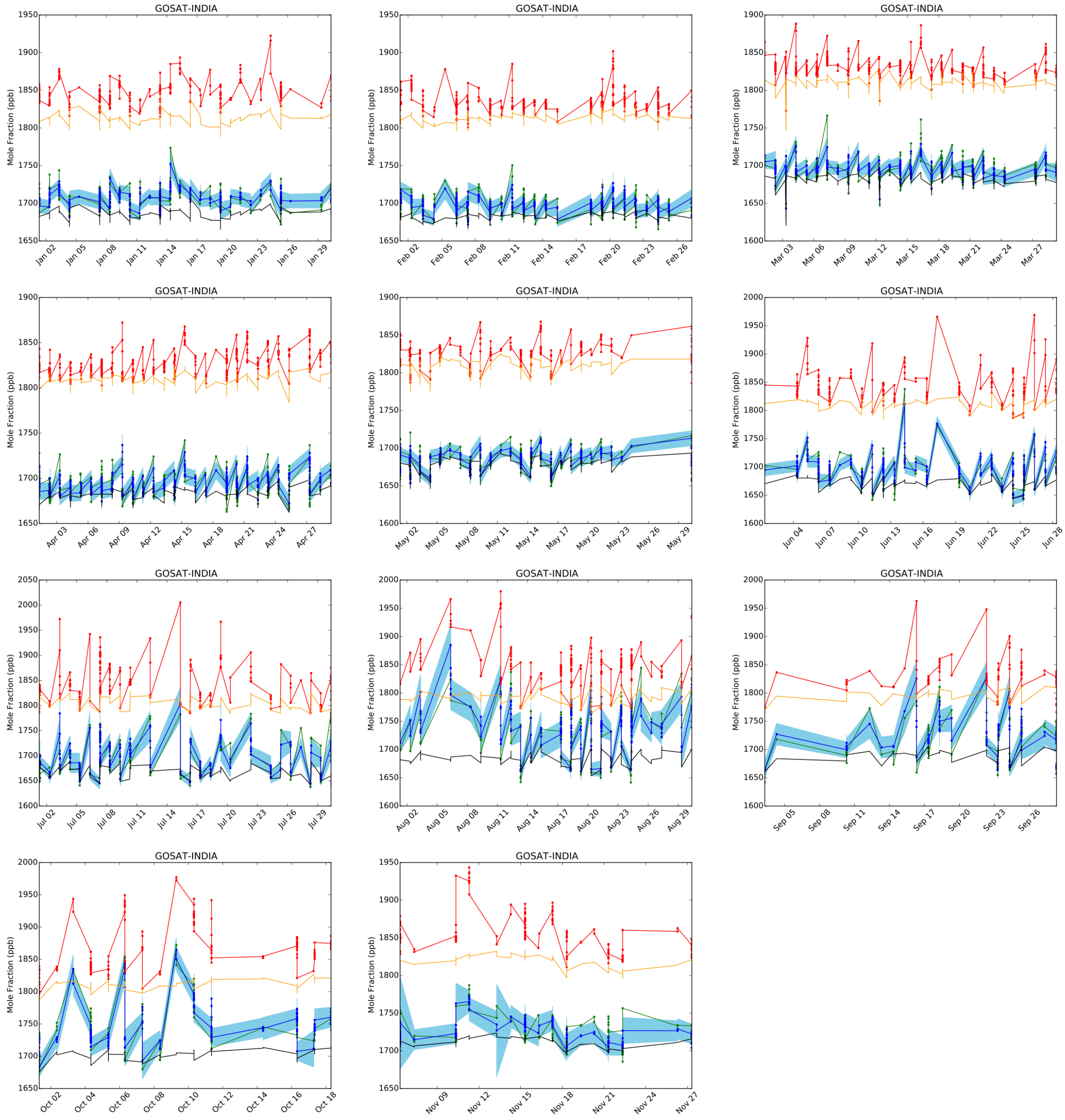

Supplementary Figure 17: GOSAT observation and model comparison (ppb) for 2014. Prior (red) and posterior (blue) mole fractions and prior (orange) and posterior (black) boundary conditions. Observations are shown in green with blue shading indicating the 5-95th percentile range of the model-measurement error derived in the inversion. Mole fractions represent the term $\boldsymbol{XCH_{4,pert}^{model}}$ in equation (6).

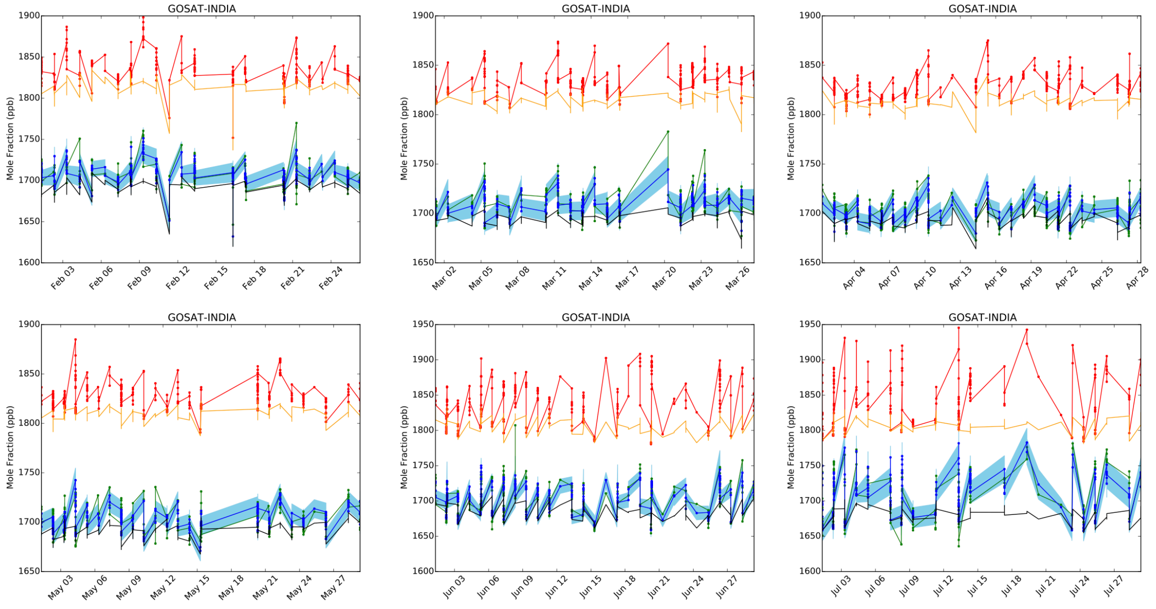

Supplementary Figure 18: GOSAT observation and model comparison (ppb) for 2015. Prior (red) and posterior (blue) mole fractions and prior (orange) and posterior (black) boundary conditions. Observations are shown in green with blue shading indicating the 5-95th percentile range of the model-measurement error derived in the inversion. Mole fractions represent the term $\boldsymbol{XCH_{4,pert}^{model}}$ in equation (6).

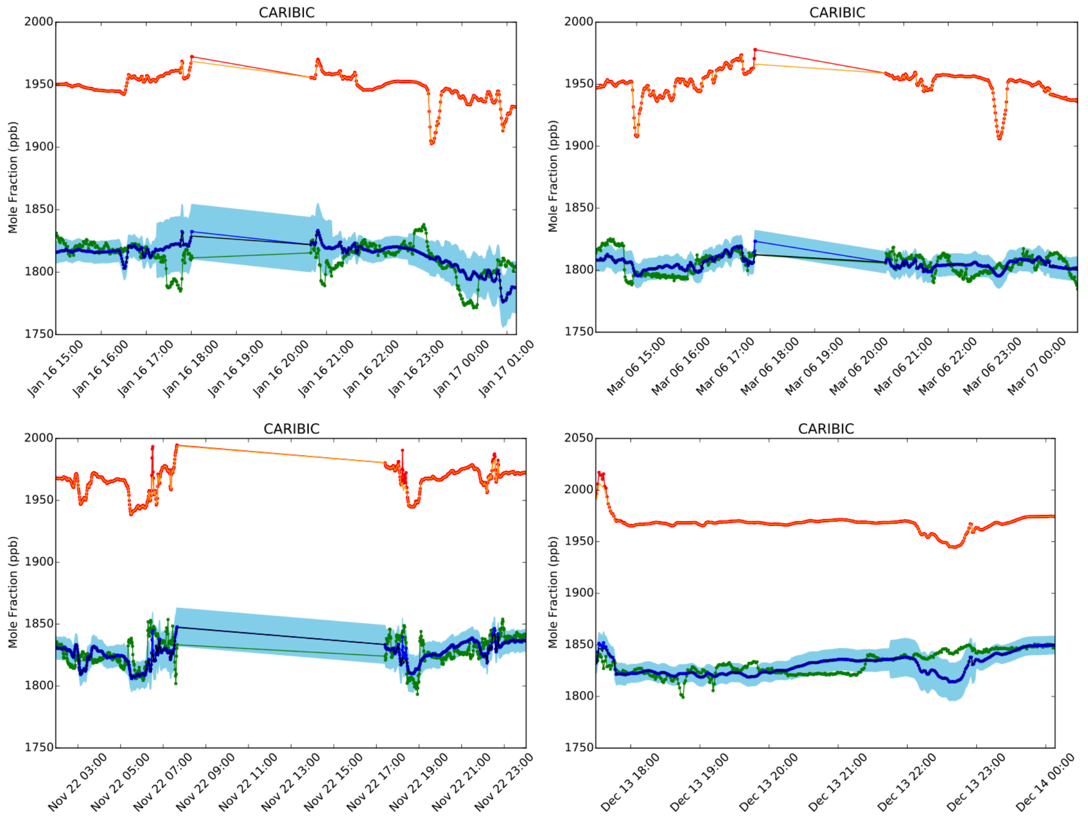

Supplementary Figure 19: CARIBIC observation and model comparison (ppb) for 2012. Prior (red) and posterior (blue) mole fractions. Observations are shown in green with blue shading indicating the 5-95th percentile range of the model-measurement error derived in the inversion. CARIBIC largely only samples boundary conditions since measurement occurs at ~30k feet and therefore these are also reflected in red/blue.

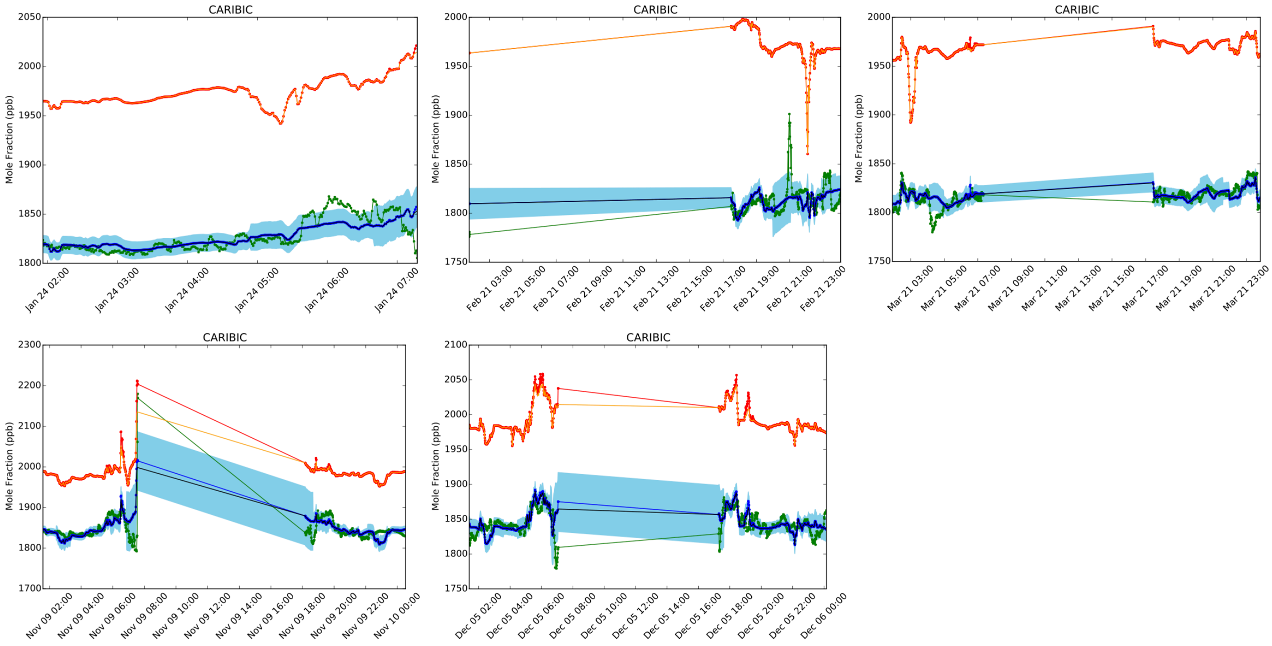

Supplementary Figure 20: CARIBIC observation and model comparison (ppb) for 2013. Prior (red) and posterior (blue) mole fractions. Observations are shown in green with blue shading indicating the 5-95th percentile range of the model-measurement error derived in the inversion. CARIBIC largely only samples boundary conditions since measurement occurs at ~30k feet and therefore these are also reflected in red/blue.

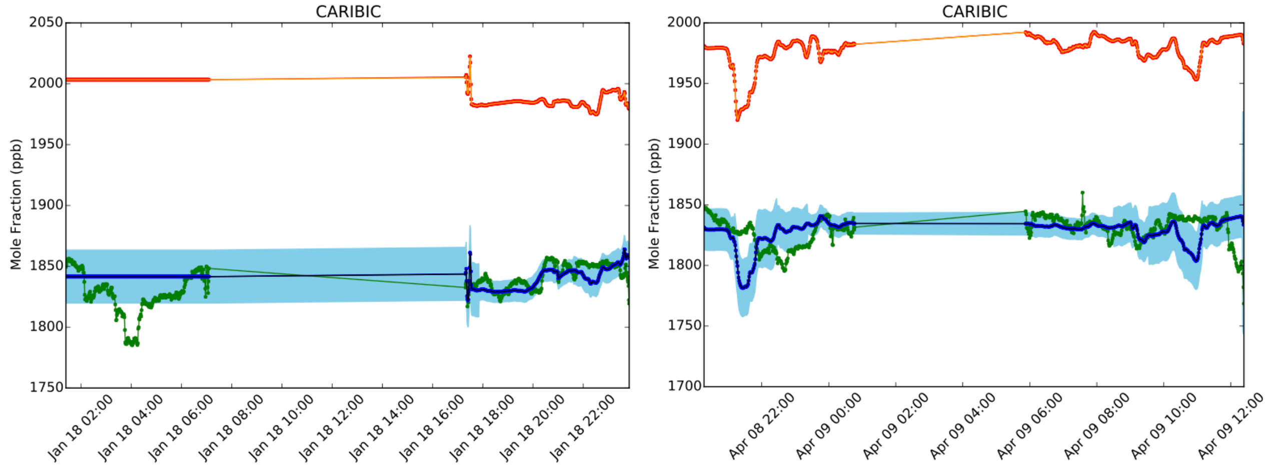

Supplementary Figure 21: CARIBIC observation and model comparison (ppb) for 2014. Prior (red) and posterior (blue) mole fractions. Observations are shown in green with blue shading indicating the 5-95th percentile range of the model-measurement error derived in the inversion. CARIBIC largely only samples boundary conditions since measurement occurs at ~30k feet and therefore these are also reflected in red/blue.

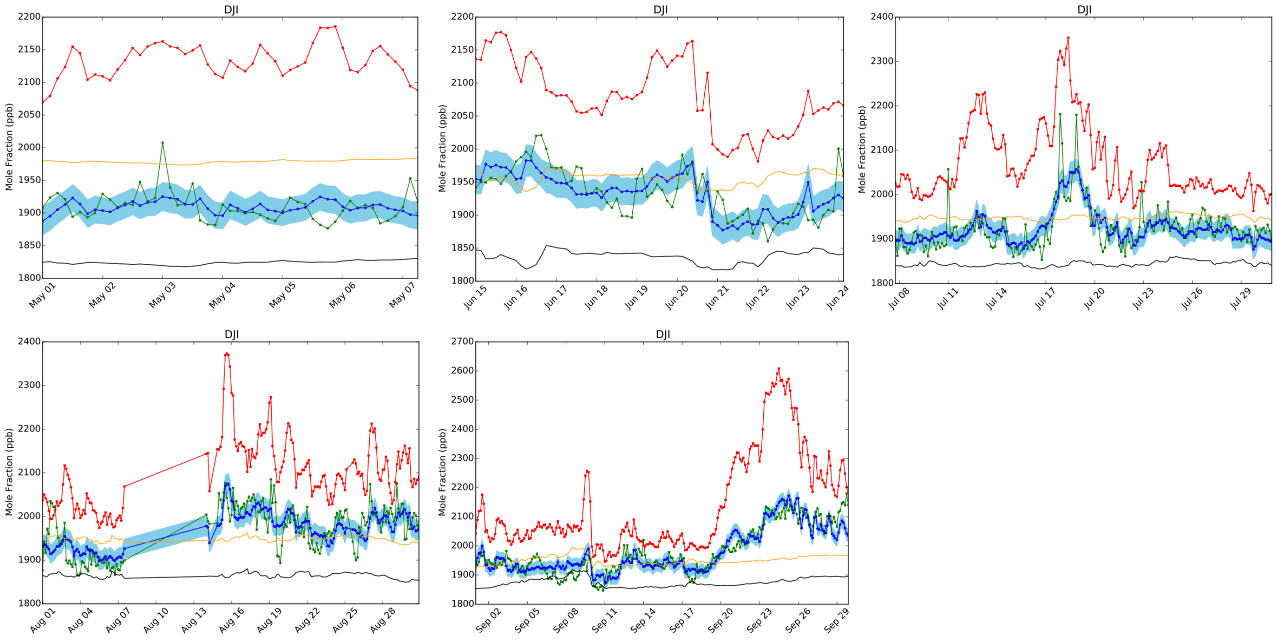

Supplementary Figure 22: Darjeeling (DJI) observation and model comparison (ppb) for 2012. Prior (red) and posterior (blue) mole fractions and prior (orange) and posterior (black) boundary conditions. Observations are shown in green with blue shading indicating the 5-95th percentile range of the model-measurement error derived in the inversion.

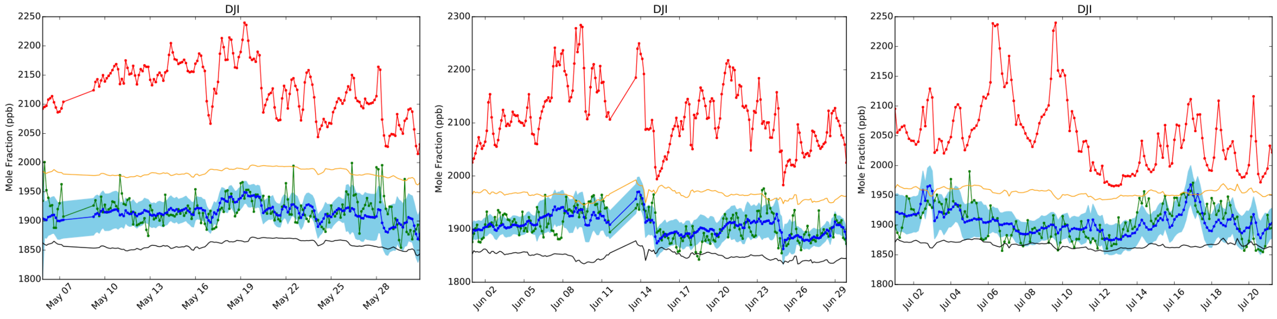

Supplementary Figure 23: Darjeeling (DJI) observation and model comparison (ppb) for 2013. Prior (red) and posterior (blue) mole fractions and prior (orange) and posterior (black) boundary conditions. Observations are shown in green with blue shading indicating the 5-95th percentile range of the model-measurement error derived in the inversion.

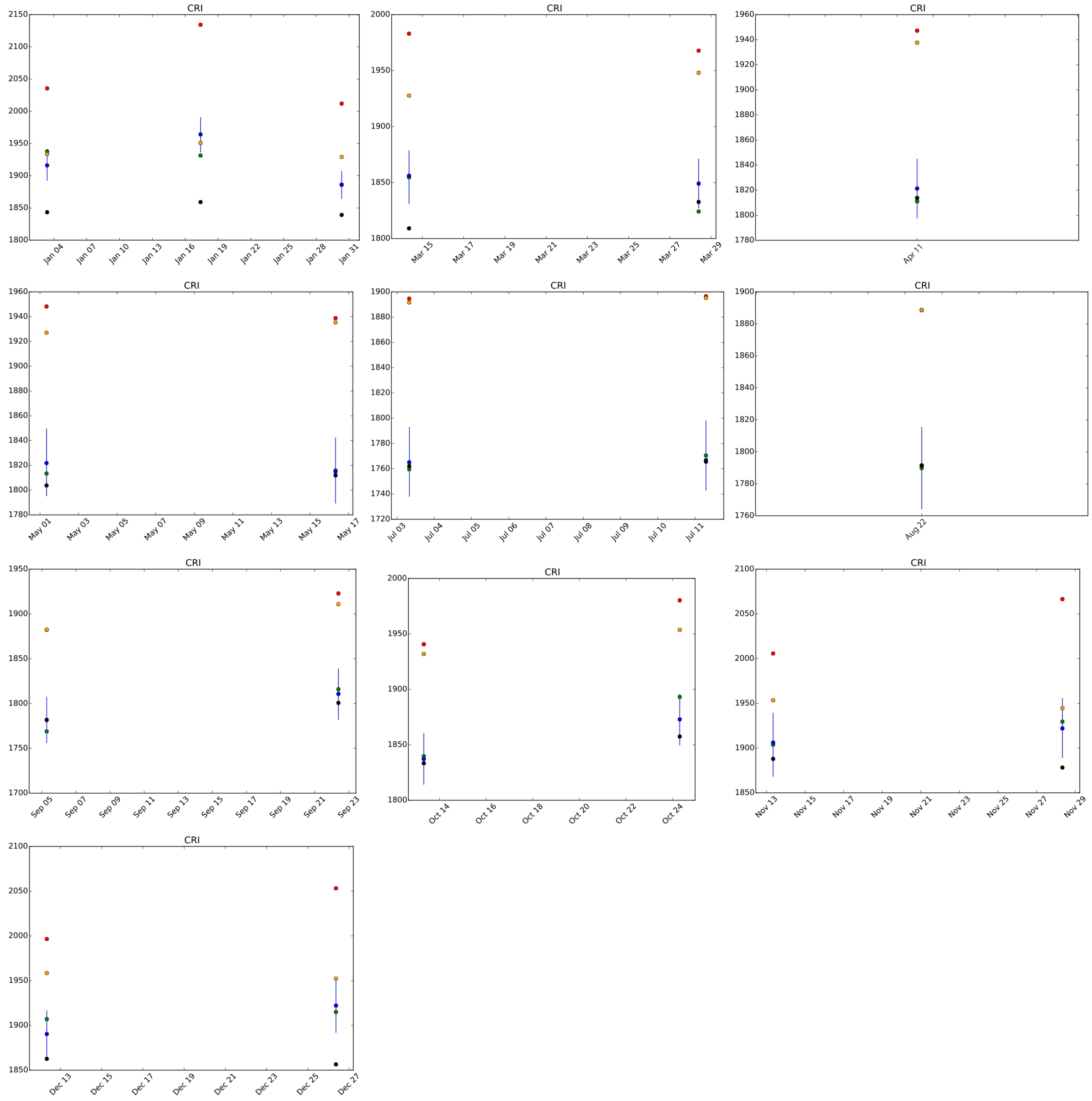

Supplementary Figure 24: Cape Rama (CRI) observation and model comparison (ppb) for 2010. Prior (red) and posterior (blue) mole fractions and prior (orange) and posterior (black) boundary conditions. Observations are shown in green with blue shading indicating the 5-95th percentile range of the model-measurement error derived in the inversion.

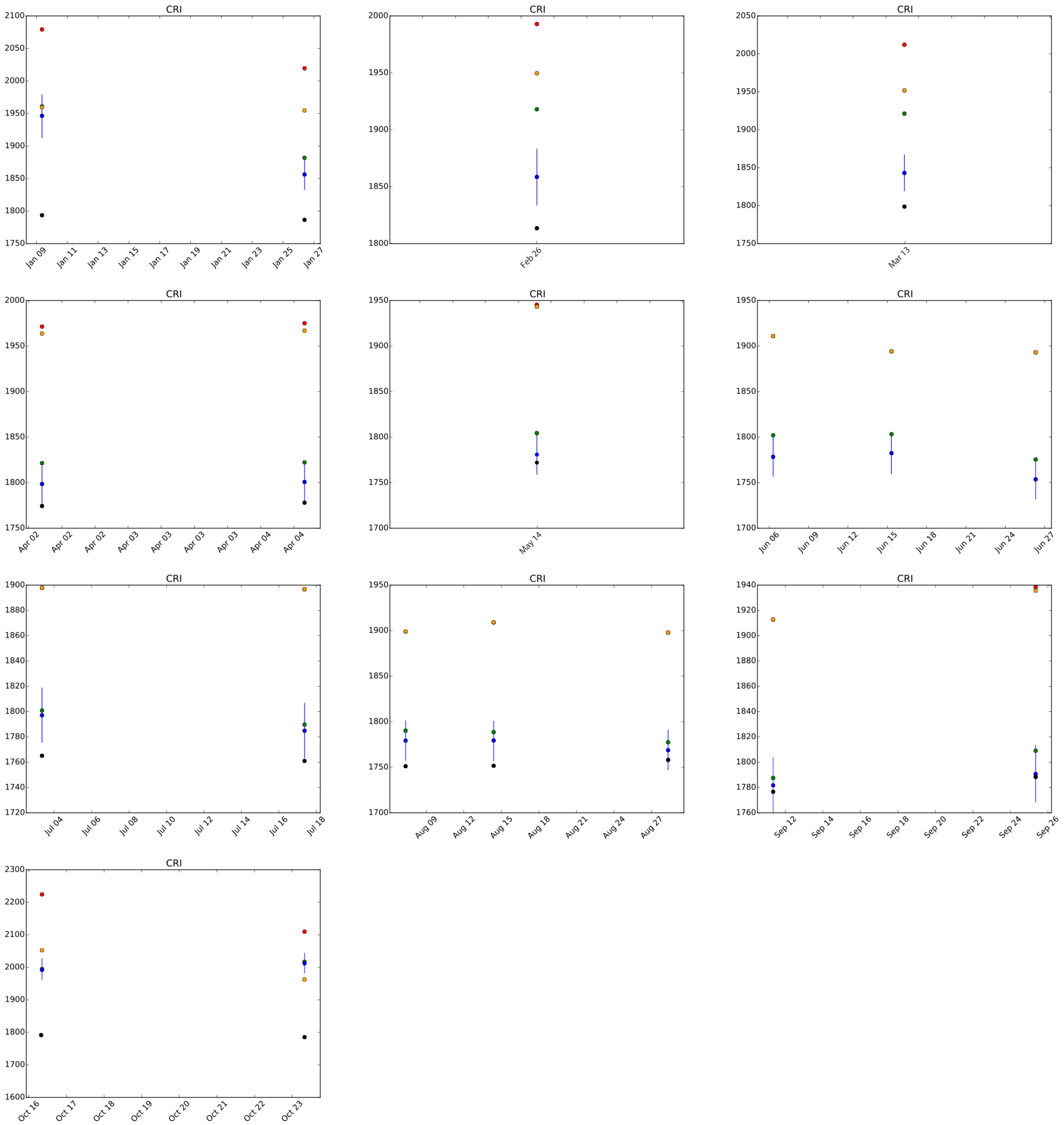

Supplementary Figure 25: Cape Rama (CRI) observation and model comparison (ppb) for 2011. Prior (red) and posterior (blue) mole fractions and prior (orange) and posterior (black) boundary conditions. Observations are shown in green with blue shading indicating the 5-95th percentile range of the model-measurement error derived in the inversion.

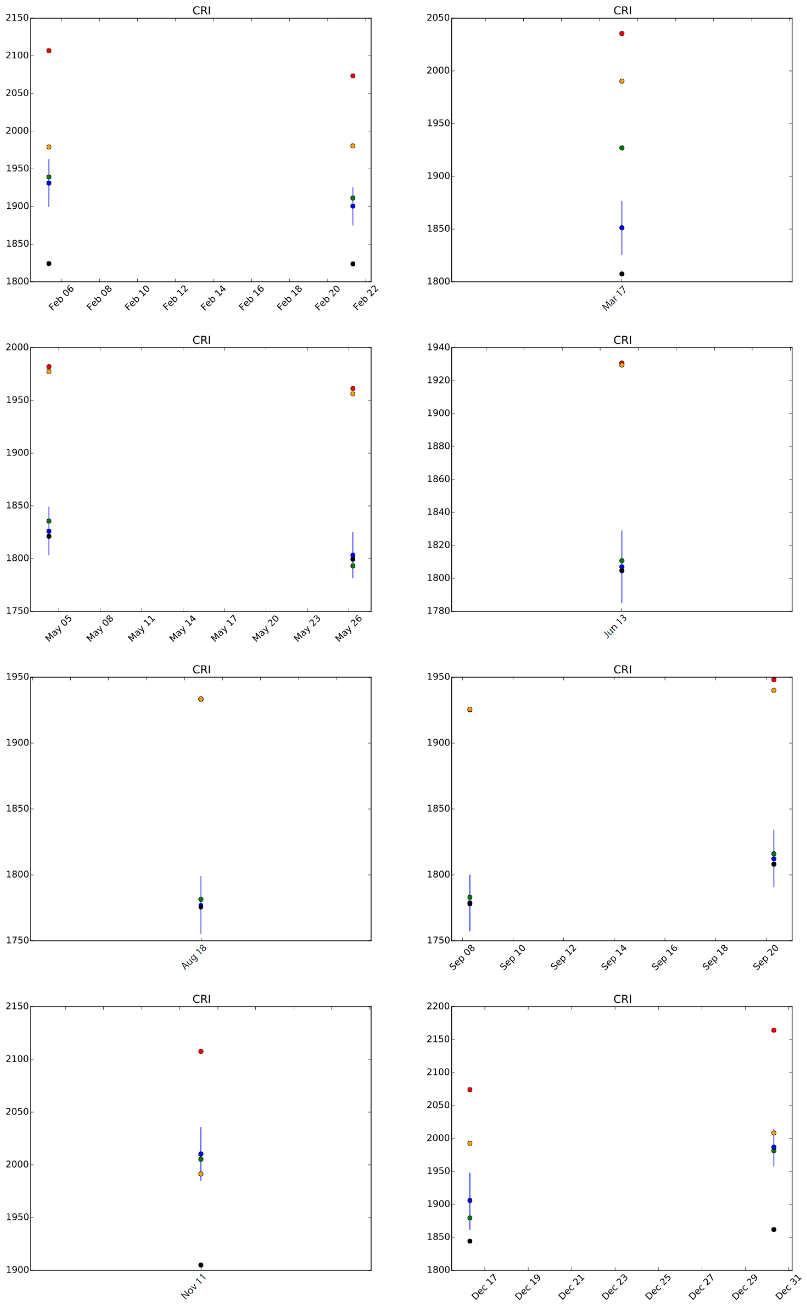

Supplementary Figure 26: Cape Rama (CRI) observation and model comparison (ppb) for 2012. Prior (red) and posterior (blue) mole fractions and prior (orange) and posterior (black) boundary conditions. Observations are shown in green with blue shading indicating the 5-95th percentile range of the model-measurement error derived in the inversion.

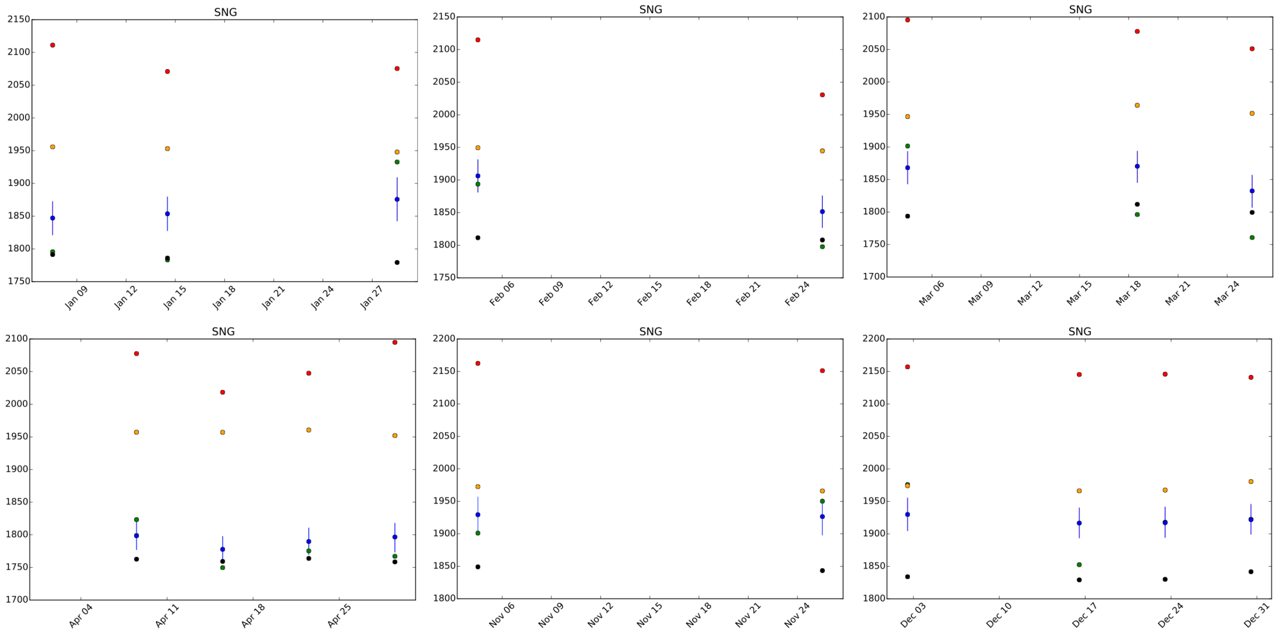

Supplementary Figure 27: Sinhagad (SNG) observation and model comparison (ppb) for 2011. Prior (red) and posterior (blue) mole fractions and prior (orange) and posterior (black) boundary conditions. Observations are shown in green with blue shading indicating the 5-95th percentile range of the model-measurement error derived in the inversion.

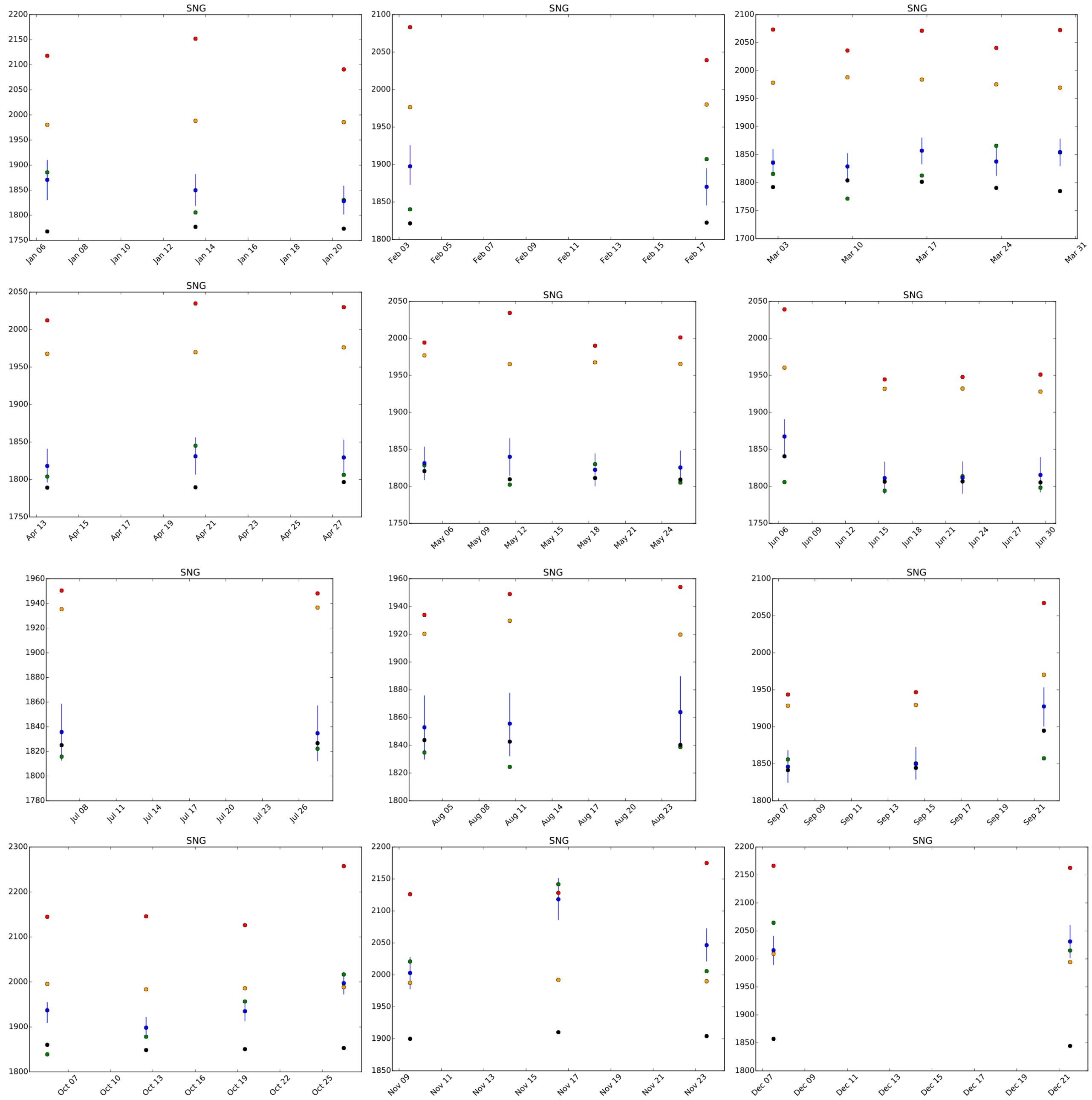

Supplementary Figure 28: Sinhagad (SNG) observation and model comparison (ppb) for 2012. Prior (red) and posterior (blue) mole fractions and prior (orange) and posterior (black) boundary conditions. Observations are shown in green with blue shading indicating the 5-95th percentile range of the model-measurement error derived in the inversion.

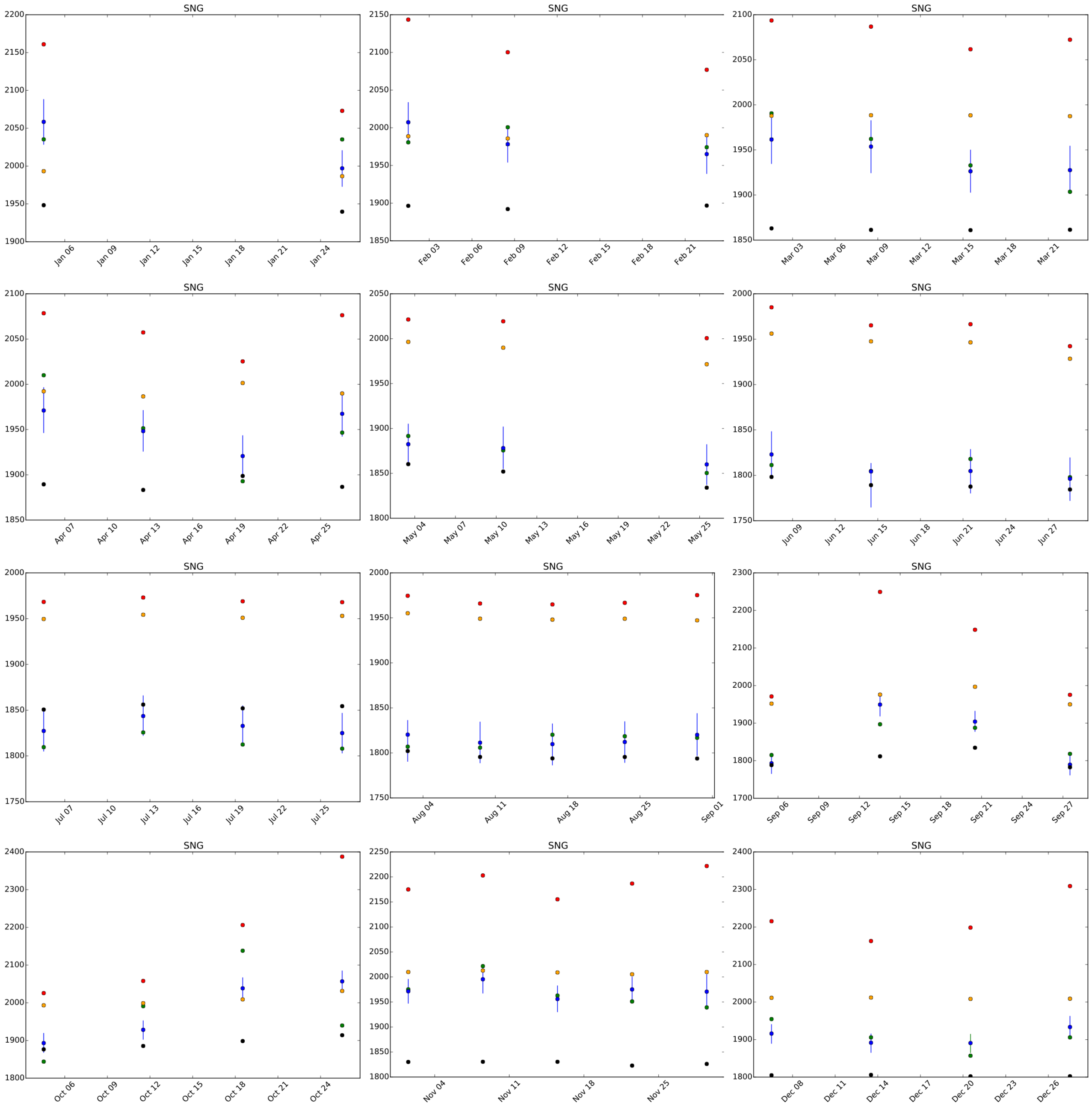

Supplementary Figure 29: Sinhagad (SNG) observation and model comparison (ppb) for 2013. Prior (red) and posterior (blue) mole fractions and prior (orange) and posterior (black) boundary conditions. Observations are shown in green with blue shading indicating the 5-95th percentile range of the model-measurement error derived in the inversion.

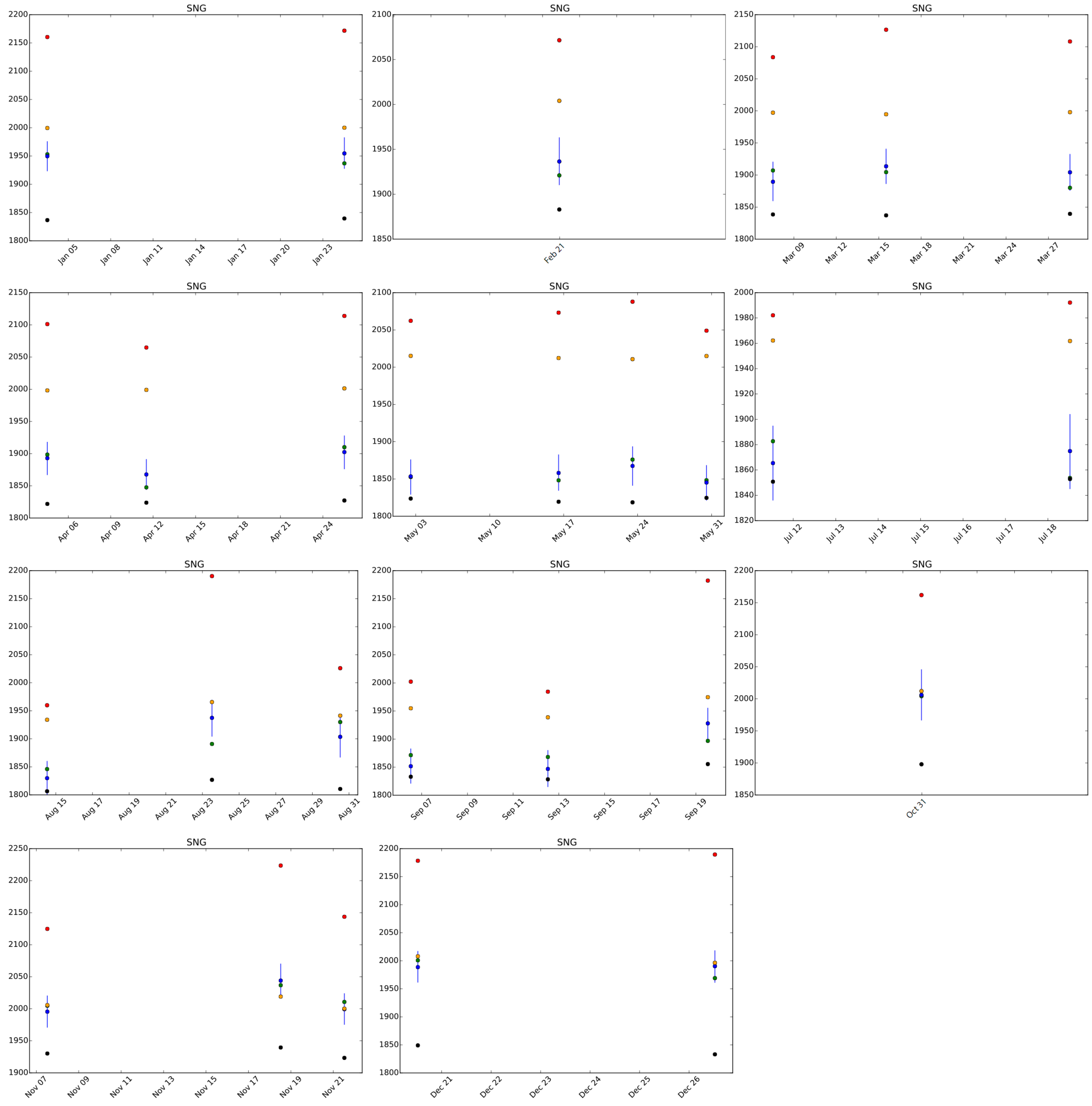

Supplementary Figure 30: Sinhagad (SNG) observation and model comparison (ppb) for 2014. Prior (red) and posterior (blue) mole fractions and prior (orange) and posterior (black) boundary conditions. Observations are shown in green with blue shading indicating the 5-95th percentile range of the model-measurement error derived in the inversion.

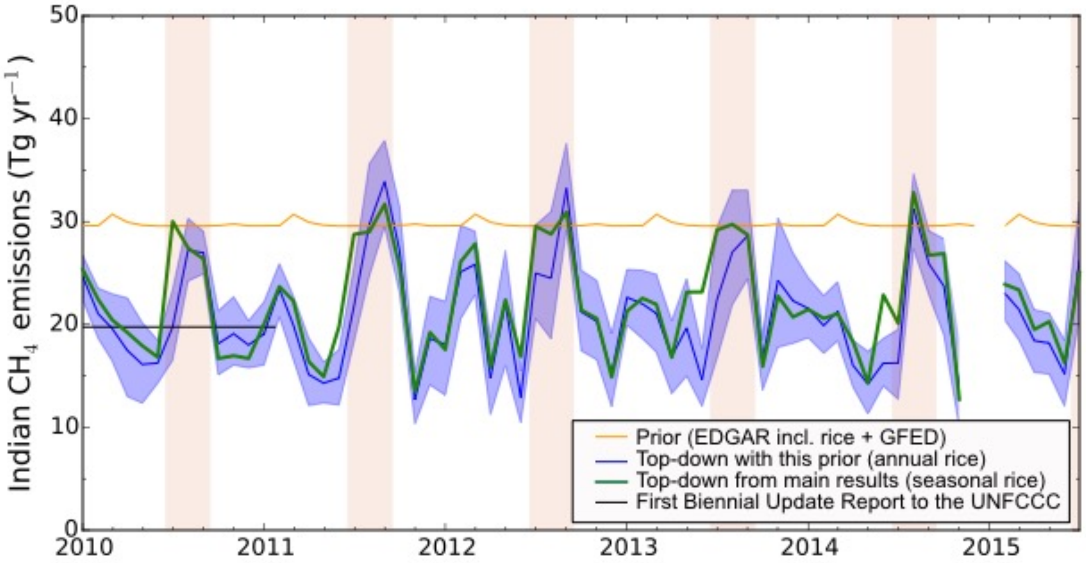

Supplementary Figure 31: Indian CH_4 emissions in Tg yr^{-1} derived (blue line) using a prior with an annual value for rice emissions (orange line). Blue shading indicates the 5th-95th percentile range of the posterior solution. Light pink bars indicates the period of the summer monsoon. The results from the main text are in green.

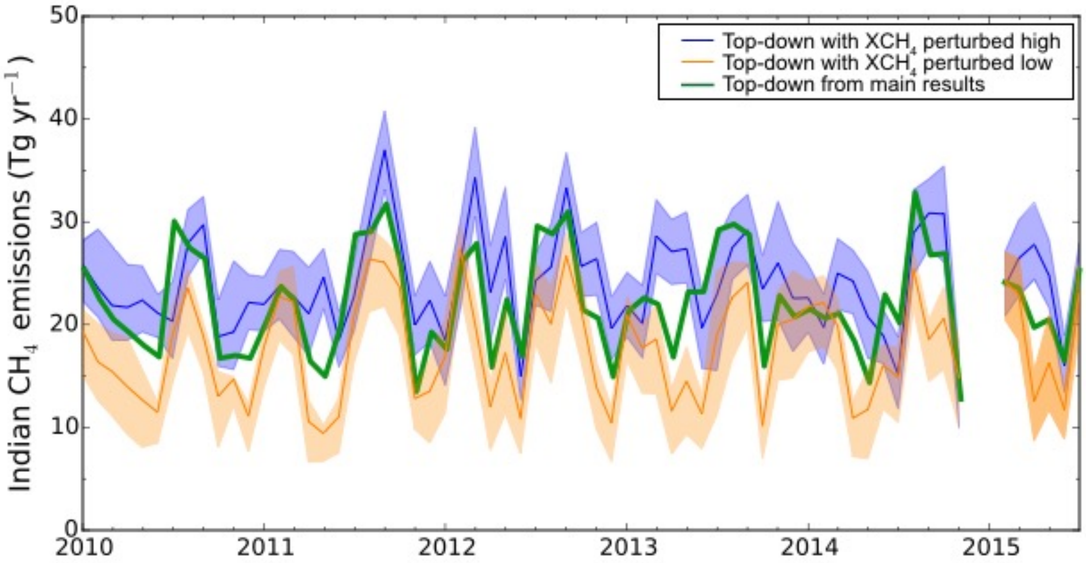

Supplementary Figure 32: Indian CH_4 emissions in Tg yr^{-1} derived with perturbed values of XCH_4. Perturbations were based on high (blue line) and low (orange line) model XCO_2 values from MACC-II, CarbonTracker and GEOS-Chem, which were then used to derive the proxy XCH_4. Blue and orange shading indicate the 5th-95th percentile range of the posterior solutions of the two perturbed scenarios. The results from the main text are in green.

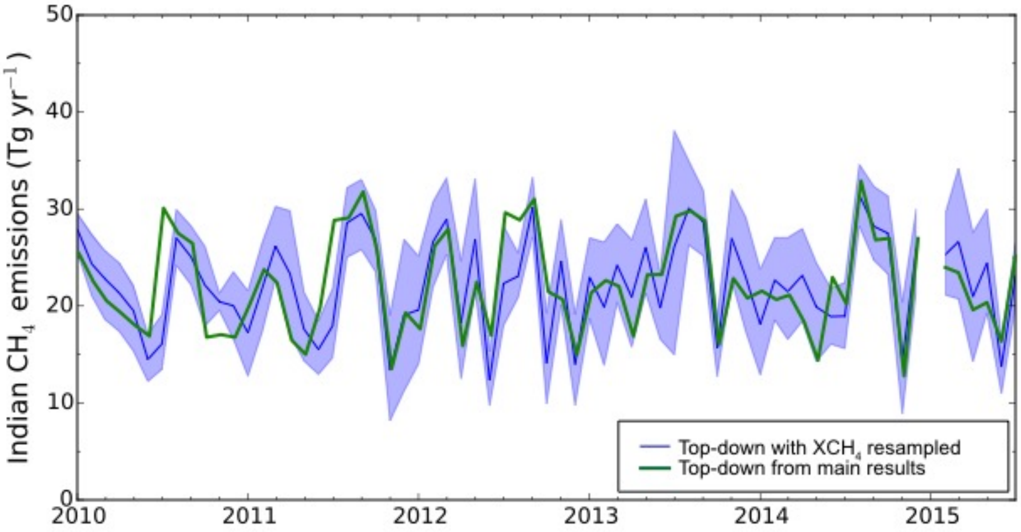

Supplementary Figure 33: Indian CH_4 emissions in Tg yr^{-1} derived with GOSAT soundings randomly resampled (blue line). In each month, 200 samples were randomly chosen. Blue shading indicates the 5th-95th percentile range of the posterior solution. The results from the main text are in green.

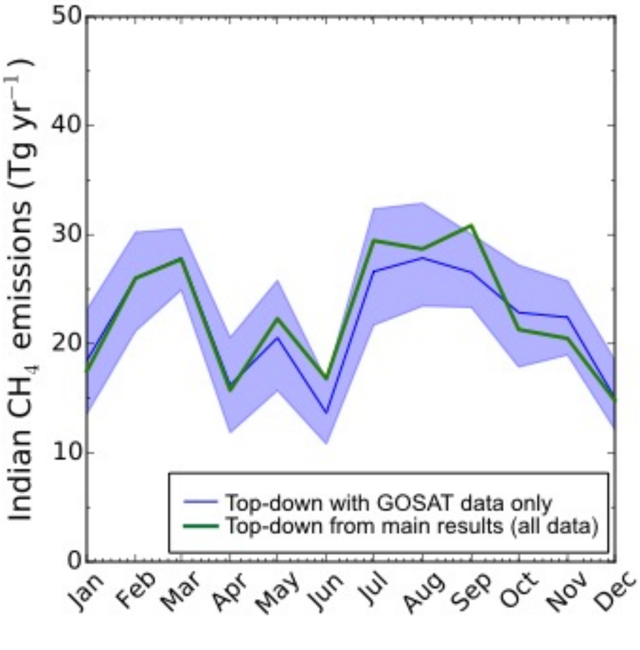

Supplementary Figure 34: Indian CH_4 emissions in Tg yr^{-1} derived using using GOSAT observations only (blue line). Blue shading indicates the 5th-95th percentile range of the posterior solution of the GOSAT only scenario. The results from the main text are in green.

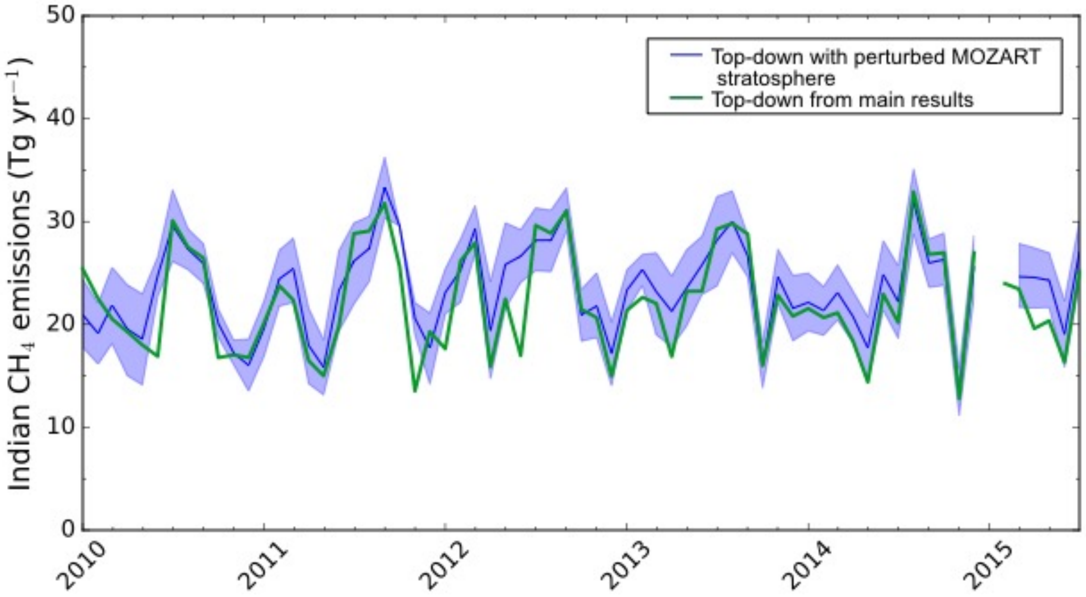

Supplementary Figure 35: Indian CH_4 emissions in Tg yr^{-1} derived with the stratosphere in the MOZART model increased by 5% (blue line). Blue shading indicates the 5th-95th percentile range of the posterior solution. The results from the main text are in green.

Supplementary Note 1:

GOSAT:
Dry air column-averaged CH_4 mole fractions, XCH_4, have been measured since 2009 in the short-wave infrared (SWIR) using the TANSO-FTS instrument on the GOSAT (Greenhouse gases Observing Satellite) satellite[2]. GOSAT measurements are valuable for flux quantification because the satellite has a repeat cycle of 3 days and has sensitivity to the near-surface. Soundings are typically separated by approximately ~100 km with diameter ~10 km. Therefore, the satellite can provide spatial and temporal coverage for regions of the world that are not well-covered by surface monitoring.

In this study, we used XCH_4 data from the University of Leicester version 6 proxy retrieval (http://www.leos.le.ac.uk/GHG/data/)[3,4] from 2010-2015. We used only those data that have been filtered for clouds and passed the quality flag and these data were averaged into 0.5° bins. Retrievals occurring over significant topography (defined as over 1000 metres above sea level) were removed from the analysis to minimize any artefacts that may arise in the retrieval algorithm due to the compressed column as well as due to errors in the transport model over these regions. Ocean glint-mode observations also were not used to avoid any systematic errors that may exist between the two modes of operation. **Supplementary Figure 7** shows time-averaged XCH_4 concentrations during each season over India. Seasons have been defined by the Indian Meteorological Department classifications: Winter (Jan-Feb), Pre-Monsoon (March-May), Monsoon/Summer (June-Sept), Post-Monsoon (Oct-Dec). Methane concentrations are highest in the Indo-Gangetic Plains (IGP) of northern India during the summer monsoon, when emissions from rice cultivation maximize, and lowest during the pre-monsoon. During winter and the pre-monsoon, concentrations are relatively uniform over the country. The seasonal changes in concentrations are due to both changes in regional emissions as well as changes in transport. Winds exhibit seasonal reversals due to the monsoon circulation.

While two methods are available (full-physics and proxy), the proxy retrieval was chosen for South Asia because we expect there to be impact from atmospheric scattering due to clouds and aerosols. XCH_4 was derived by multiplying the measured XCH_4/XCO_2 ratio from a non-scattering atmosphere by a model XCO_2 field (equation 1). Because XCO_2 varies much less in the atmosphere than does XCH_4, it is assumed that the errors in XCO_2 are of much smaller magnitude than the errors due to incompletely quantified atmospheric scattering in the full-physics retrieval. This is particularly important for regions of high aerosol loading and/or cloud cover.[5]

$$XCH_4^{proxy} = \frac{XCH_4}{XCO_2} \times XCO_2^{model} \tag{1}$$

The main limitation of the proxy method is that it relies on accuracy of the model XCO_2 field, which has assimilated surface CO_2 observations using a chemical transport model. Proxy XCH_4 has been used in a variety of global and regional studies[6–12] and has been validated against surface, aircraft and TCCON measurements[3,4,13,14].

Firstly, we investigated the range of XCO_2 between the three models, which are found in version 6 of the GOSAT product. **Supplementary Figure 8** shows the percentage range of these models relative to the median model (which varies for each retrieval). The smallest and largest differences, as a percentage, occur in winter and summer, respectively. The maximum difference, which affects a small subset of retrievals, is on the order of 1%. To assess the sensitivity of national emissions to the range of XCO_2 fields, we have repeated our inversions by scaling each XCH_4 retrieval up and down by the percentage difference in Supplementary Figure 8 and these results are discussed further in Supplementary Note 3

We have also compared MACC-II model output[15] against CO_2 mole fractions measured at Cape Rama, India (CRI) and on the CARIBIC aircraft **(Supplementary Figure 9)**. It is important to note that because CO_2 measurements are sparse in India and this analysis is limited to two datasets, this comparison is presented to show the potential impact of this error, rather than to account for it. Because the inversion results will only be sensitive to spatial gradients in the CO_2 model error (uniform differences would be absorbed into boundary conditions and offset terms), there is insufficient information to know what effect this error would have on the inversion results. The main difference between CRI data and model CO_2 occurs during the post-monsoon and winter. While the model simulates the minimum of the seasonal cycle in November, this is not evident in the CRI data. At these times, the model is as much as 8% lower than the observed value. All other times of year appear consistent between model and observations. This discrepancy could be due to the resolution of the model or due to the prior flux field used. The CARIBIC data shows that the model could be biased slightly low (<0.1%) but this is less pronounced than the CRI comparison. Because the aircraft samples in the upper troposphere, spatial gradients are much smaller than at the surface and therefore, it is expected that the differences between the model and observations will not be as large. This data comparison suggests that results from October-January should be interpreted with greater caution. However, it should be noted that the effect on the column will be much less extreme than what is shown at the surface. Our results during October-January do not show any extreme changes relative to other times of the year.

Previous studies have also found systematic errors between GOSAT retrievals and ground-based FTIR measurements from TCCON (Total Carbon Column Observing Network) but TCCON data is not available in South Asia[3,16]. Systematic errors between GOSAT and TCCON have typically been less than 5 ppb. In addition, studies have found biases between GOSAT and model derived column mole fractions[7], which could additionally be due to systematic errors in the chemical transport model. These errors have exhibited a latitudinal dependence on the order of 20 ppb, peaking in the tropics. Many inverse studies account for a systematic error in the inversion between satellite and calibrated data, which we also incorporate into our inverse method.

Surface stations:

We used data from three surface stations (Darjeeling, India, DJI; Cape Rama, India, CRI; Sinhagad, India, SNG). DJI (88.25°E,27.03°N, 2200 m.a.s.l.) is located in the Himalayas in the eastern part of India. DJI was an in situ station with high-frequency CH_4 observations on a gas chromatography-flame ionization detector (GC-FID) between 2012-2013[17]. Measurements were calibrated on the Tohoku University calibration scale but converted to the NOAA-2004 scale using an inter-calibration factor of 1.0003[18]. For this study, measurements were averaged into 3-hour periods and only used for the period May-September when the meteorology of the region is conducive for sampling large-scale regional emissions rather than more localized sources[17]. CRI is located on the western coast in Goa (73.83°E, 15.08°N, 60 m.a.s.l), where weekly flask measurements were made until 2013. Measurements were made on a GC-FID and calibrated on the NOAA-2004 scale[19]. SNG is located near Pune in the western Ghats region of India (73.75°E, 18.35°N, 1600 m.a.s.l). Flasks were measured weekly through 2014 on a GC-FID and calibrated on the NOAA-2004 scale[20].

CARIBIC:

We used data from the CARIBIC passenger aircraft program, including 24 winter (Jan-Mar, Nov-Dec) flights over South Asia (http://www.caribic-atmospheric.com). Summer flights over South Asia were discontinued after 2008 but have been used to estimate monsoon-related biogenic production of CH_4 from South Asia[21]. These flights took place either from Frankfurt, Germany to Chennai, India or Frankfurt to Bangkok, Thailand. Methane mole fractions were measured in flight using a Los Gatos Research Fast Greenhouse Gas Analyzer at 1 Hz frequency. The precision with 10s averaging is approximately 1 ppb[22] and measurements were calibrated using whole air samples

on the NOAA-2004 calibration scale[23]. To minimize the effect of contaminated samples from airports, air was only sampled at altitudes with pressure below 650 hPa. For this study, measurements were averaged into 1 minute periods.

Supplementary Note 2:

Model uncertainties derived through the hierarchical inversion method were derived for each 5-day period per surface site or per 5-degree latitude bin for satellite/aircraft observations. Average model uncertainties on GOSAT retrievals were found to be 14.0 ppb, with the largest uncertainties in July - September (mean model error of 20.4 ppb) and the lowest in January-April (mean of 9.0 ppb). These uncertainties are reflected in the national emissions presented in (manuscript Fig. 1A) which shows the smallest and largest uncertainties at these times. There are two possible reasons for the larger errors during the summer: firstly, emissions and therefore concentrations above background are higher so absolute uncertainties are also larger and secondly, the summer period coincides with the Indian summer monsoon, a period of intense and deep convection, which is likely to not be as well-represented in the model. Offsets between satellite and calibrated data were also derived along with an analysis of the systematic errors associated with incorrect specification of the CO_2 model used to derive the proxy XCH_4 data used in this study.

Offsets between GOSAT and surface/aircraft observations were also estimated in the inversion, assuming that the surface/aircraft data were `unbiased' through anchoring to a calibration scale. We estimated one offset value for the domain per month but this could be extended to represent a physical process such as bias in the XCO_2 field. On average, summertime offsets (defined as $y_{gosat, obs} - y_{gosat, mod}$) were +38.6 ppb (largely based on GOSAT and DJI data). Wintertime offsets, in contrast (largely based on GOSAT and CARIBIC), were -8.1 ppb. The offset that is estimated is a combination of offset due to the observations themselves as well as any offset due to the model and both of these are expected to vary seasonally. The model component also includes any error in the assumption of the prior model mole fractions being correct above *maxlev*. We have found that changes in the representation of the mole fractions above the maximum level in NAME (i.e. assuming either prior model mole fractions or assuming a constant mole fraction between *maxlev*-20) had little effect on the emissions that were derived and were largely absorbed into the offset term. The changes to the offset term in these two scenarios suggests that a significant component of the offset term could be due to the representation of the upper atmosphere in the model. Supplementary Note 3 discusses the effect of using GOSAT data alone versus the full set of observations and the effect of changes to the model stratosphere.

Supplementary Note 3:

Five sensitivity studies were performed to assess the effect on the top-down results of (1) the prior emissions; (2) XCO_2 model error; (3) GOSAT tracks; (4) differences between using the full set of surface, aircraft, and satellite data and satellite data alone; (5) errors in the model stratosphere.

(1) A sensitivity study was performed to assess the influence that the prior seasonal cycle had on the estimated emissions. For the prior of this sensitivity study, we used EDGAR v4.2FT2010 (including rice), GFED v3.1 and natural sources and did not use the seasonally varying rice prior used in the main results. Though the prior did not contain a summertime monsoon signature, the same summertime maximum emerged, showing that the results are largely data-driven and not heavily influenced by the prior (**Supplementary Figure 31**). Emissions derived with this prior largely fall within the uncertainties of the top-down emissions presented in the main results.

(2) We assessed the effect of errors in the model XCO_2 used to derive the proxy XCH_4 on derived emissions. **Supplementary Figure 32** shows national emissions derived using perturbed values of XCH_4 based on differences between three CO_2 models and inversion frameworks. Each retrieval was perturbed high or low based on the model CO_2 range for that retrieval. In this way, differences in the spatial distribution of the model errors (**Supplementary Figure 8**) were accounted for. While the median emissions of the base case occasionally lie outside of the uncertainties in the perturbed cases, the uncertainties overlap. These findings suggest that the variability between the three CO_2 models does play a role in the emissions derived for the region, however, it should be noted, that all three models are based on a similar set of underlying CO_2 data, which are from global networks and not from South Asia. Furthermore, it is not expected that all retrievals would necessarily be biased consistently in the same direction, so these results reflect the extreme. Larger biases could result from inaccuracies in model CO_2 compared to observed CO_2, as discussed above. These findings suggest that the uncertainties quantified using this methodology should be extended to account for systematic errors to more completely capture all sources of error.

(3) We assessed the effect of randomly resampling 200 measurements from the GOSAT observations each month. This was done to determine whether the spatial tracks of the GOSAT soundings could impart any systematic bias into the results. **Supplementary Figure 33** shows that the emissions derived with the random resampling are consistent with the results provided in the main text. This provides evidence that the spatial tracks of GOSAT are not introducing artefacts into the results.

(4) We assessed the effect of using the full set of satellite, aircraft and surface data (with offsets estimated and accounted for) versus using GOSAT data alone. This was performed for the year 2012 when all data was available. This was done is determine whether results are influenced by the varying amounts of data available for different time periods. **Supplementary Figure 34** shows little difference in national emissions derived using satellite data alone and emissions derived using all available data over 2012 when all sites were operational. The difference between the two is insignificant, suggesting that the effect of offsets have been accounted for in the derived boundary conditions because they are generally spatially uniform across India.

(5) We assessed the effect of errors in the model's simulation of the stratosphere based on findings from Saad et al., 2016[26]. This study found that errors in the model simulation of stratospheric CH_4 could lead to biases in XCH_4, which could result in errors in derived surface fluxes. To assess the impact of this error, we perturbed the prior boundary conditions by 5% (approximately 100 ppb at the upper levels, as shown by Figure 5 in Saad et al., 2016[26]). **Supplementary Figure 35** shows that the differences in derived fluxes lie within the uncertainties presented in our base case. We propose that these small differences are because this is a regional inversion and any errors in stratosphere-troposphere exchange will be corrected each month from observations in that month. Any errors in the model boundary conditions, which were used as a <u>prior</u> in the inversion, will be minimized as offsets and gradients to the prior field are estimated in the inversion.

Supplementary References:

1. Yan, X., Akiyama, H., Yagi, K. & Akimoto, H. Global estimations of the inventory and mitigation potential of methane emissions from rice cultivation conducted using the 2006 Intergovernmental Panel on Climate Change Guidelines. *Global Biogeochem. Cycles* **23,**

GB2002 (2009).
2. Kuze, A., Suto, H., Nakajima, M. & Hamazaki, T. Thermal and near infrared sensor for carbon observation Fourier-transform spectrometer on the Greenhouse Gases Observing Satellite for greenhouse gases monitoring. *Appl. Opt.* **48,** 6716 (2009).
3. Parker, R. *et al.* Methane observations from the Greenhouse Gases Observing SATellite: Comparison to ground-based TCCON data and model calculations. *Geophys. Res. Lett.* **38,** L15807 (2011).
4. Parker, R. J. *et al.* Assessing 5 years of GOSAT Proxy XCH_4 data and associated uncertainties. *Atmos. Meas. Tech.* **8,** 4785–4801 (2015).
5. Frankenberg, C., Meirink, J. F., van Weele, M., Platt, U. & Wagner, T. Assessing methane emissions from global space-borne observations. *Science* **308,** 1010–1014 (2005).
6. Bergamaschi, P. *et al.* Atmospheric CH_4 in the first decade of the 21st century: Inverse modeling analysis using SCIAMACHY satellite retrievals and NOAA surface measurements. *J. Geophys. Res. Atmos.* **118,** 7350–7369 (2013).
7. Fraser, A. *et al.* Estimating regional methane surface fluxes: the relative importance of surface and GOSAT mole fraction measurements. *Atmos. Chem. Phys.* **13,** 5697–5713 (2013).
8. Wecht, K. J. *et al.* Spatially resolving methane emissions in California: constraints from the CalNex aircraft campaign and from present (GOSAT, TES) and future (TROPOMI, geostationary) satellite observations. *Atmos. Chem. Phys.* **14,** 8173–8184 (2014).
9. Turner, A. J. *et al.* Estimating global and North American methane emissions with high spatial resolution using GOSAT satellite data. *Atmos. Chem. Phys.* **15,** 7049–7069 (2015).
10. Turner, A. J. *et al.* A large increase in U.S. methane emissions over the past decade inferred from satellite data and surface observations. *Geophys. Res. Lett.* **43,** 2218–2224 (2016).
11. Alexe, M. *et al.* Inverse modelling of CH_4 emissions for 2010-2011 using different satellite retrieval products from GOSAT and SCIAMACHY. *Atmos. Chem. Phys.* **15,** 113–133 (2015).
12. Cressot, C. *et al.* On the consistency between global and regional methane emissions inferred from SCIAMACHY, TANSO-FTS, IASI and surface measurements. *Atmos. Chem. Phys.* **14,** 577–592 (2014).
13. Dils, B. *et al.* The Greenhouse Gas Climate Change Initiative (GHG-CCI): comparative validation of GHG-CCI SCIAMACHY/ENVISAT and TANSO-FTS/GOSAT CO_2 and CH_4 retrieval algorithm products with measurements from the TCCON. *Atmos. Meas. Tech.* **7,** 1723–1744 (2014).
14. Webb, A. J. *et al.* CH 4 concentrations over the Amazon from GOSAT consistent with in situ vertical profile data. *J. Geophys. Res. Atmos.* **121,** 11,006-11,020 (2016).
15. Chevallier, F. *et al.* CO_2 surface fluxes at grid point scale estimated from a global 21 year reanalysis of atmospheric measurements. *J. Geophys. Res. Atmos.* **115,** D21307 (2010).
16. Pandey, S. *et al.* Inverse modeling of GOSAT-retrieved ratios of total column CH_4 and CO_2 for 2009 and 2010. *Atmos. Chem. Phys.* **16,** 5043–5062 (2016).
17. Ganesan, A. L. *et al.* The variability of methane, nitrous oxide and sulfur hexafluoride in Northeast India. *Atmos. Chem. Phys.* **13,** 10633–10644 (2013).
18. Dlugokencky, E. J. *et al.* Conversion of NOAA atmospheric dry air CH4 mole fractions to a gravimetrically prepared standard scale. *J. Geophys. Res.* **110,** D18306 (2005).
19. Bhattacharya, S. K. *et al.* Trace gases and CO_2 isotope records from Cabo de Rama, India. *Curr. Sci.* **97,** 1336–1344 (2009).
20. Tiwari, Y. K., Vellore, R. K., Ravi Kumar, K., van der Schoot, M. & Cho, C.-H. Influence of monsoons on atmospheric CO_2 spatial variability and ground-based monitoring over India. *Sci. Total Environ.* **490,** 570–578 (2014).
21. Baker, A. K. *et al.* Estimating the contribution of monsoon-related biogenic production to methane emissions from South Asia using CARIBIC observations. *Geophys. Res. Lett.* **39,** L10813 (2012).

22. Dyroff, C. *et al.* Tunable diode laser in-situ CH_4 measurements aboard the CARIBIC passenger aircraft: Instrument performance assessment. *Atmos. Meas. Tech.* **7,** 743–755 (2014).
23. Schuck, T. J. *et al.* Greenhouse gas relationships in the Indian summer monsoon plume measured by the CARIBIC passenger aircraft. *Atmos. Chem. Phys.* **10,** 3965–3984 (2010).
24. Manning, A. J., O'Doherty, S., Jones, A. R., Simmonds, P. G. & Derwent, R. G. Estimating UK methane and nitrous oxide emissions from 1990 to 2007 using an inversion modeling approach. *J. Geophys. Res.* **116,** D02305 (2011).
25. Ganesan, A. L. *et al.* Quantifying methane and nitrous oxide emissions from the UK and Ireland using a national-scale monitoring network. *Atmos. Chem. Phys.* **15,** 6393–6406 (2015).
26. Saad, K. M. *et al.* Seasonal variability of stratospheric methane: Implications for constraining tropospheric methane budgets using total column observations. *Atmos. Chem. Phys.* **16,** 14003–14024 (2016).
